# DCYTB is a predictor of outcome in breast cancer that functions via iron-independent mechanisms

**DOI:** 10.1186/s13058-017-0814-9

**Published:** 2017-03-07

**Authors:** David J. Lemler, Miranda L. Lynch, Lia Tesfay, Zhiyong Deng, Bibbin T. Paul, Xiaohong Wang, Poornima Hegde, David H. Manz, Suzy V. Torti, Frank M. Torti

**Affiliations:** 1 Department of Molecular Biology and Biophysics, University of Connecticut Health Center, Farmington, CT 06030 USA; 2 Center for Quantitative Medicine, University of Connecticut Health Center, Farmington, CT 06030 USA; 3 Department of Pathology, University of Connecticut Health Center, Farmington, CT 06030 USA; 4 School of Dental Medicine, University of Connecticut Health Center, Farmington, CT 06030 USA; 5 Department of Medicine, University of Connecticut Health Center, Farmington, CT 06030 USA; 6 Present address: Department of Molecular Biomedical Sciences, North Carolina State University, CVM Research Building 474, Raleigh, NC 27695 USA; 7 Present address: Statistical Sciences Group CCS-6, Los Alamos National Laboratory, Los Alamos, NM 87545 USA

**Keywords:** DCYTB, Iron, Breast, Cancer, Prognosis, Survival, Cancer therapy, Focal adhesion

## Abstract

**Background:**

Duodenal cytochrome b (DCYTB) is a ferrireductase that functions together with divalent metal transporter 1 (DMT1) to mediate dietary iron reduction and uptake in the duodenum. DCYTB is also a member of a 16-gene iron regulatory gene signature (IRGS) that predicts metastasis-free survival in breast cancer patients. To better understand the relationship between DCYTB and breast cancer, we explored in detail the prognostic significance and molecular function of DCYTB in breast cancer.

**Methods:**

The prognostic significance of DCYTB expression was evaluated using publicly available microarray data. Signaling Pathway Impact Analysis (SPIA) of microarray data was used to identify potential novel functions of DCYTB. The role of DCYTB was assessed using immunohistochemistry and measurements of iron uptake, iron metabolism, and FAK signaling.

**Results:**

High DCYTB expression was associated with prolonged survival in two large independent cohorts, together totaling 1610 patients (cohort #1, *p* = 1.6e-11, n = 741; cohort #2, *p* = 1.2e-05, n = 869; log-rank test) as well as in the *Gene expression-based Outcome for Breast cancer Online* (GOBO) cohort (*p* < 1.0e-05, n = 1379). High DCYTB expression was also associated with increased survival in homogeneously treated groups of patients who received either tamoxifen or chemotherapy. Immunohistochemistry revealed that DCYTB is localized on the plasma membrane of breast epithelial cells, and that expression is dramatically reduced in high-grade tumors. Surprisingly, neither overexpression nor knockdown of DCYTB affected levels of ferritin H, transferrin receptor, labile iron or total cellular iron in breast cancer cells. Because SPIA pathway analysis of patient microarray data revealed an association between DCYTB and the focal adhesion pathway, we examined the influence of DCYTB on FAK activation in breast cancer cells. These experiments reveal that DCYTB reduces adhesion and activation of focal adhesion kinase (FAK) and its adapter protein paxillin.

**Conclusions:**

DCYTB is an important predictor of outcome and is associated with response to therapy in breast cancer patients. DCYTB does not affect intracellular iron in breast cancer cells. Instead, DCYTB may retard cancer progression by reducing activation of FAK, a kinase that plays a central role in tumor cell adhesion and metastasis.

**Electronic supplementary material:**

The online version of this article (doi:10.1186/s13058-017-0814-9) contains supplementary material, which is available to authorized users.

## Background

Iron has been implicated in both the initiation and progression of cancer. Due to its ability to catalyze the formation of oxygen free radicals, iron can facilitate DNA damage and lead to potentially mutagenic changes in DNA [1]. Iron can also act as a tumor growth factor, potentiating the growth of numerous tumors, including breast tumors, in animal models [2, 3]. Consistent with these laboratory studies, epidemiologic studies have linked excess iron and cancer [4–7]. For example, subjects with increased levels of circulating iron are at increased risk of cancer [8–10], and conversely, subjects who have undergone phlebotomy for iron reduction are at decreased cancer risk [7].

The major mechanism of iron import in both normal and malignant cells is the transferrin/transferrin receptor endocytic pathway. Two molecules of ferric iron bound to transferrin are endocytosed upon transferrin receptor binding. Iron is released in the acidified endosome, reduced, and imported into the cytosol, where it enters a low molecular weight, metabolically active labile iron pool (LIP). Excess iron in the cytosol is stored in ferritin or exported via the iron exporter, ferroportin [11]. Other mechanisms of iron import include uptake of heme, ferritin, and import of siderophore-bound iron by proteins such as the secreted glycoprotein Lipocalin 2 (LCN2, NGAL), [12–15].

In the duodenum, where uptake of dietary iron occurs, the mechanism of iron import involves duodenal cytochrome b (DCYTB) [16–18]. Dietary iron is largely present in an oxidized form (ferric iron, Fe^+3^). DCYTB acts as a ferrireductase, reducing ferric iron to ferrous iron to permit iron uptake by divalent metal transporter 1 (DMT1). Identified in 2001 [16], DCYTB is a member of the cytochrome b561 protein family of di-heme, transplasma membrane electron transporters [19, 20]. Reduction of iron by DCYTB is pH-dependent and ascorbate-dependent in duodenal enterocytes [16–18, 21], but ascorbate-independent in bronchial epithelial cells [22]. Copper is also a substrate for reduction by DCYTB, a reaction that occurs in a pH-independent, ascorbate-dependent manner [18]. Additionally, DCYTB expression has been shown to maintain extracellular levels of ascorbate [23].

Cancer cells exhibit an enhanced requirement for iron compared to their normal counterparts. To meet the increased metabolic demand for iron, breast and other cancer cells frequently increase expression of the iron importer transferrin receptor [24–26]. Alternatively or additionally, cancer cells suppress expression of the iron efflux protein ferroportin [27]. Although retained iron is sequestered in ferritin, this nevertheless results in an increase in labile iron [27–29].

Measurements of the expression of genes of iron metabolism are strong predictors of patient prognosis. For example, breast cancer patient microarray data demonstrate that increased transferrin receptor expression [30–32] or decreased ferroportin expression in breast tumors are associated with poor prognosis [27]. Tumoral expression of LCN2 is also associated with poor prognosis and increased metastasis in breast cancer [33, 34].

To ascertain which components of iron metabolism most influence breast cancer prognosis, our group studied the association of 61 “iron” genes with breast cancer patient outcome [32]. From these analyses, an “iron gene regulatory signature” was derived, consisting of 16 genes whose expression best predicted breast cancer patient outcome. Of these 16 genes, expression of duodenal cytochrome b (DCYTB, CYBRD1, CYB561A2) was the most significantly associated with distant metastasis-free survival (DMFS), with high expression (values above the mean) associated with a reduced hazard ratio of 0.6 (*p* = 1.8e-07). Since DCYTB facilitates iron import, its association with improved outcome was surprising. The expression of this gene in the breast was also unanticipated, since its best-known function involves uptake of dietary iron.

We therefore sought to understand in greater depth the nature of the association of DCYTB with breast cancer, and to explore the role of DCYTB in the breast. We first expanded our assessment of the ability of DCYTB to predict patient survival and response to therapy utilizing large, independent gene expression datasets obtained from breast cancer patients. We then investigated whether DCYTB expression influenced iron homeostasis in malignant breast cells. Our results indicate that DCYTB expression is strikingly associated with patient outcome and response to therapy. However, we found that DCYTB does not affect intracellular iron in breast cancer cells. Rather, DCYTB inhibits FAK activation and cell adhesion. These results uncouple DCYTB from iron metabolism in breast cancer tissue and provide an explanation for the paradoxical association between increased DCYTB expression and favorable prognosis in breast cancer patients.

## Results

### DCYTB as a prognostic indicator of breast cancer

#### Expression of DCYTB predicts metastasis/relapse-free survival

**Fig. 1:**
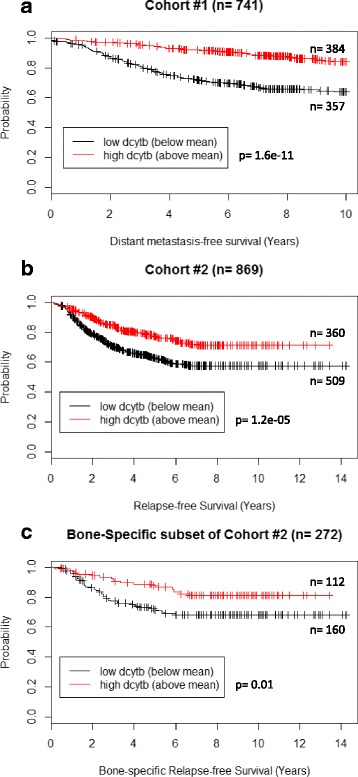
High DCYTB expression is associated with increased recurrence-free survival in breast cancer. Kaplan-Meier analysis of breast cancer patient microarray data subsetted into high and low DCYTB expression groups (above and below the mean). **a** Cohort #1 distant metastasis-free survival (*p* = 1.6e-11, n = 741, log-rank test); **b** cohort #2 relapse-free survival (both local and distant) (*p* = 1.2e-05, n = 869, log-rank test); **c** subgroup of cohort #2 (GSE2034) bone-specific relapse-free survival (*p* = 0.01, n = 272, log-rank test). *DCYTB* duodenal cytochrome b

We first examined the prognostic significance of DCYTB when considered as a single gene rather than as part of the larger IRGS gene signature [32]. Analysis of the combined cohort of 741 breast cancer patients that was used in the design of the IRGS [32] (herein termed cohort #1), revealed that high DCYTB expression (values above the mean) was an excellent overall predictor of distant metastasis-free survival (*p* = 1.6e-11, n = 741, log-rank test; Fig. 1a).

We then validated and expanded our results using additional datasets not included in cohort #1, which we combined into a new cohort of 869 patients (cohort #2; see “Methods”). To construct this cohort, we selected all larger datasets (n > 100) with sufficient events to meaningfully separate patients by outcome (Table 1). Datasets that did not meet these criteria (e.g., GSE19615 [35], TCGA) were excluded. In cohort #2, DCYTB expression above the mean was again dramatically associated with increased relapse-free survival (RFS) (*p* = 1.2e-05, n = 869; log-rank test; Fig. 1b). One of the datasets used to construct cohort #2 contained information on bone-specific RFS; analysis of this subgroup (n = 272) further revealed that DCYTB expression was associated with bone-specific RFS (Fig. 1c). Consistent with the association of high DCYTB with favorable prognosis, we further observed that expression of DCYTB was higher in tumors that expressed estrogen receptor (ER+) than in ER- tumors (Additional file 1: Figure S1). Additionally, DCYTB expression decreased with increased tumor grade (Additional file 1: Figure S2).

**Table 1:** Characteristics of patients and samples used in microarray analysis

Name	Number	Event^d^ (%)	Median recurrence-free survival (years)	Median follow-up (years)	Mean age, years (SD)	ER +/-	LN +/-	Her2	PR +/-	Grade 1/2/3	Normal tissue	Reference
Cohort #1^a^	741	167 (22)	7.8	8.7	60.8 (12.5)	643/89	364/358	NA	NA/NA	153/314/188	NA	Miller et al. [32]
Cohort #2^b^	869	251 (29)	4.3	4.5	51(11.1)	486/276	342/426	6	239/250	43/209/309	14^c^	
GSE25055	303	63 (21)	2.4	2.6	50 (10.3)	170/133	217/86	4	140/157	19/113/149	NA	Hatzis et al. [40]
GSE25065	193	42 (22)	3.2	3.7	49.2 (10.6)	120/72	125/68	2	99/93	13/59/107	NA	Hatzis et al. [40]
GSE2034	272	100 (37)	7.2	8.6	54 (12)	196/76	0/272	NA	NA/NA	NA	NA	Wang et al. [73]
GSE42568	101	46 (46)	5.3	6.2	58.6 (11.6)	65/33	58/43	NA	NA/NA	11/37/53	14^c^	Clarke et al. [74]

Complete datasets were not available for all patients. Discrepancies between total number of patients in cohorts and the number of patients evaluated based on individual characteristics (ER status, LN status, Her2, PR, grade) are due to missing data in each of these categories

*ER* estrogen receptor, *LN* lymph node, *Her2* human epidermal growth factor 2, *PR* progesterone receptor, *NA* not applicable

^a^Constructed from data in caArray: mille-00271; GEO: GSE1456, GSE6532, GSE9195

^b^Constructed from data in GEO: GSE25055, GSE25065, GSE2043, GSE42568

^c^Normal tissue samples are not included in the number of patients (n)

^d^Cohort #1 events are DMFS (distant metastasis-free survival). All other cohorts are RFS (recurrence-free survival)

We used the gene expression-based outcome for breast cancer online database (GOBO [36]) to assess the effects of DCYTB expression in another large combined cohort. Analysis of this dataset similarly indicates that high DCYTB expression is associated with increased DMFS (*p* < 0.00001, n = 1379, Additional file 1: Figure S3a).

**Fig. 2:**
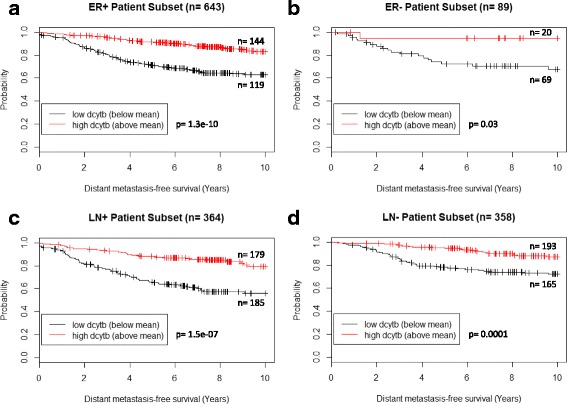
DCYTB predicts outcome independent of ER and LN status. Kaplan-Meier analysis of patients in cohort #1 subsetted by high and low DCYTB expression and **a** ER+ (*p* = 1.3e-10, n = 643), **b** ER- (*p* = 0.03, n = 89), **c** LN+ (*p* = 1.5e-07, n = 364), **d** LN- (*p* = 0.0001, n = 358). *DCYTB* duodenal cytochrome b, *ER* estrogen receptor, *LN* lymph node

We next tested whether DCYTB expression was predictive in both estrogen receptor-positive (ER+) and ER- cohorts. Kaplan-Meier survival analysis of cohort #1 indicated that DCYTB significantly predicted DMFS independently of estrogen receptor status (*p* = 1.3e-10 and *p* = 0.03, log-rank test, Fig. 2a,b). Similarly, analysis of cohort #2 revealed that high DCYTB expression was associated with increased relapse-free survival of both ER+ and ER- patients (*p* = 0.004 and *p* = 0.01, log-rank test, Additional file 1: Figure S4a,b).

We also tested whether DCYTB distinguished outcome in patients whose disease remained confined to the breast (LN-) and patients whose disease had spread to adjacent lymph nodes (LN+). In cohort #1, DCYTB expression predicted DMFS in both LN+ and LN- patients (*p* ≤ 0.0001, log-rank test Fig. 2c,d). The association of elevated DCYTB expression with prolonged relapse-free survival was also observed in LN+ and LN- patients of cohort #2 (*p* = 0.02 and *p* = 0.0001, log-rank test, Additional file 1: Figure S4c,d).

Multivariate analysis of all patients in cohorts #1 and #2 was then used to determine whether DCYTB was an independent predictor of outcome. When characteristics of patients’ primary tumors (i.e. ER status, size, and grade) were considered in a multivariate Cox proportional hazards analysis, DCYTB remained an independent predictor of outcome (*p* = 0.03, n = 612, Cox PH, Table 2). Thus DCYTB is an independent predictor of outcome in patients whose disease remains confined to the breast. The presence of disease in adjacent lymph nodes (LN status) is an indicator of propensity to metastatic dissemination. In this combined cohort, DCYTB expression was not independent of LN status when LN status was included in the model (*p* = 0.25, n = 424, Cox PH, Table 2), suggesting that DCYTB expression and LN status may convey somewhat overlapping information – i.e., a propensity toward disease dissemination. In contrast, in the larger GOBO dataset, DCYTB remained an independent predictor of outcome when all variables, including LN status, were included in the model (*p* = 0.01, n = 571, Additional file 1: Figure S3b). In aggregate, these results indicate that high DCYTB expression is associated with a more favorable prognosis in breast cancer patients.

**Table 2:** Univariate and multivariate regression analysis of combined cohorts 1 and 2

	Univariate			Multivariate^ac^ (LN status excluded)			Multivariate^bc^ (LN status included)		
Covariates	Cox *P* value ^*^	HR	95% CI	Cox *P* value ^*^	HR	95% CI	Cox *P* value^*^	HR	95% CI
DCYTB	1.11E-07	0.79	0.73–0.87	0.03	0.84	0.71–0.98	0.25	0.90	0.75–1.08
Size	0.41	1.00	0.99–1.01	0.02	1.01	1.00–1.02	0.05	1.01	0.99–1.02
Grade 2	1.20E-05	2.34	1.60–3.42	3.90E-03	2.07	1.26–3.38	0.01	1.97	1.17–3.34
Grade 3	2.53E-11	3.64	2.50–5.32	7.51E-04	2.53	1.48–4.35	0.01	2.29	1.27–4.15
ER status	4.14E-07	0.56	0.45–0.69	0.59	0.89	0.57–1.37	0.36	0.79	0.47–1.32
LN status	7.14E-05	1.53	1.24–1.89	-	-	-	0.01	1.55	1.09–2.21

*LN* lymph node, *HR* hazard ratio, *CI* confidence interval, *DCYTB* duodenal cytochrome b, *ER* estrogen receptor,

*Likelihood ratio test *P* value

^a^612 patients had complete clinical annotation for size, grade, age and ER status

^b^464 patients had complete clinical annotation for size, grade, ER and LN status

^c^ER status was not significant in the multivariate analysis, possibly due to collinearity with grade in this dataset or to criteria used in assembling cohort #2 (in particular, the requirement for a relatively high (>20%) event rate, which may have enriched for studies with fewer ER+/LumA tumors)

#### DCYTB expression correlates with the better prognosis breast cancer molecular subtypes

**Fig. 3:**
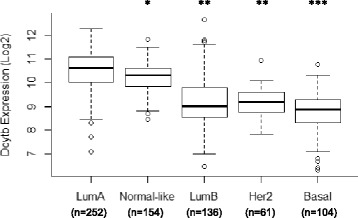
Increased DCYTB expression in molecular subtypes with better outcome in cohort #1. DCYTB expression in each breast cancer molecular subtype of cohort #1. Luminal A, n = 252; Normal-like, n = 154; Luminal B, n = 136; Her2, n = 61; Basal, n = 104. ^*^ *p* ≤ 0.0028 vs LumA, ^**^ *p* ≤ 2.8e-15 vs LumA and Normal-like cohorts, ^***^ *p* ≤ 0.0027 vs all other cohorts. *DCYTB* duodenal cytochrome b

We then investigated the expression of DCYTB within breast cancer intrinsic molecular subtypes. These subtypes can be used to divide patients into prognostic subgroups based on gene expression profiles [37, 38]. When cohort #1 was divided into intrinsic subtypes, the expected prognostic associations with patient outcomes were observed [37, 39]: Luminal A and Normal-like demonstrated better outcomes, and Luminal B, Basal and Her2 had less favorable survival (Additional file 1: Figure S5). We found that DCYTB expression was higher in subtypes with more favorable prognoses (Fig. 3). Thus, Luminal A subtype had significantly higher DCYTB expression than all other subtypes (*p* ≤ 0.0028, pairwise *t* test). Similarly, Normal-like subtype had significantly higher DCYTB expression than all other subtypes with less favorable prognosis (*p* ≤ 2.8e-15, pairwise *t* test). Basal subtype, which is associated with a poorer breast cancer prognosis, had significantly reduced DCYTB expression compared to all other subtypes (*p* ≤ 0.0027, pairwise *t* test). Subtype information was also available for a subset of patients from cohort #2 (Additional file 1: Figure S6a). Similar to what we observed in cohort #1, cohort #2 patients with Luminal A subtype had significantly more DCYTB expression compared to Luminal B, Her2 and Basal subtypes and the Normal-like subtype was significantly increased compared to Luminal B and Basal (Additional file 1: Figure S6b). Thus, high DCYTB expression is associated with subtypes that have better outcome.

#### Expression of DCYTB is associated with response to therapy

**Fig. 4:**
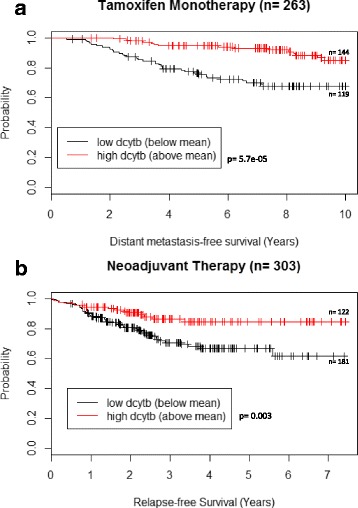
DCYTB predicts treatment outcome in tamoxifen and chemotherapy-treated cohorts. **a** Kaplan-Meier analysis of ER+ patients in cohort #1 who received tamoxifen monotherapy subsetted by high and low DCYTB expression, *p* = 5.7e-05, n = 263, log-rank test. **b** Kaplan-Meier analysis of patients who were ERBB2- (Her2-) and either ER+ or ER- and had been treated with taxane-anthracycline neoadjuvant chemotherapy (and tamoxifen if ER+) followed by surgery (GSE25055) [40], *p* = 0.003, n = 303, log-rank test. *DCYTB* duodenal cytochrome b

Finally, we asked whether DCYTB expression was associated with response to therapy. To address this question, we first examined a subset of ER+, LN- patients from cohort #1 that were histologically similar and had been treated with tamoxifen monotherapy (n = 263) [32]. DCYTB expression identified patients with improved DMFS in this group (*p* = 5.7e-05, log-rank test; Fig. 4a). To determine whether DCYTB also separated patients treated with chemotherapy, we examined a subset of cohort #2. This group consisted of 303 patients who were ERBB2- (Her2-) and either ER+ or ER- and had been treated with taxane-anthracycline neoadjuvant chemotherapy (and tamoxifen if ER+) followed by surgery (GSE25055) [40]. We found that DCYTB identified patients with improved RFS in this group (*p* = 0.003, log-rank test, Fig. 4b). Thus DCYTB can identify subgroups with different outcomes within homogeneously treated patient groups who have received either chemotherapy or hormone therapy.

### DCYTB expression and localization in normal and malignant breast tissue

We next investigated the level of DCYTB in normal and malignant breast tissue. This analysis was restricted to cohort #2 because only cohort #2 contained normal breast samples. We observed that normal breast tissue exhibited significantly higher levels of DCYTB mRNA than malignant tissue (Additional file 1: Figure S7). Data from the Cancer Genome Atlas (TCGA) representing 1100 tumors and 112 normal controls similarly revealed an increase in DCYTB expression in normal breast tissue (Additional file 1: Figure S7B).

We then assessed the cellular distribution and localization of DCYTB in breast tissue using immunohistochemical analysis of a tissue microarray containing 75 breast cancer cases and nonmalignant controls. Our first objective was to assess whether DCYTB was limited to breast epithelial cells or was present in immune, endothelial, adipose or other cell types that constitute tumor tissue. We also expected to gain information on the potential function of DCYTB by assessing its intracellular distribution. In the duodenum, where DCYTB functions in iron import, DCYTB is localized to the brush border, on the surface of the enterocyte [16]. In both esophageal carcinoma and in normal and malignant colon, however, DCYTB is located in the membrane of intracellular vesicles [41, 42]. Other members of the cytochrome b561 family, which function in vesicular catecholamine synthesis and lysosomal degradation, are expressed in the membrane of intracellular organelles [20, 43, 44].

**Fig. 5:**
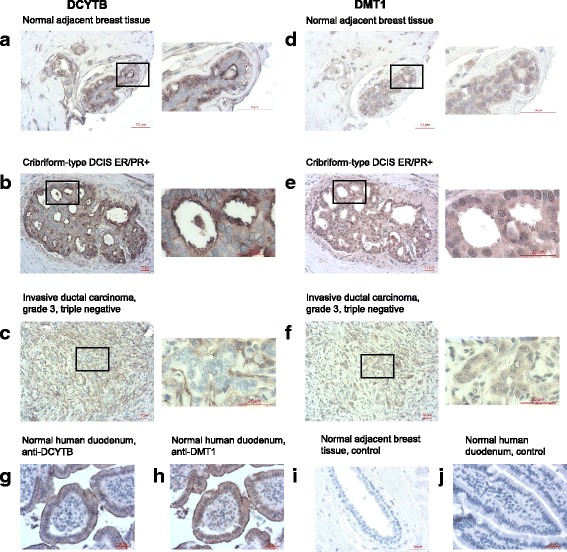
Tissue expression of DCYTB and DMT1. Representative images of immunohistochemical staining of a breast tissue microarray (×20 magnification) and de-identified duodenal tissue from UConn Health Center Department of Pathology. **a**, **d** Normal adjacent breast tissue; **b**, **e** cribriform-type DCIS ER/PR+; **c**, **f** invasive ductal carcinoma, grade 3, triple negative; **g**, **h** normal human duodenum, ×40; **i** normal adjacent breast tissue, control stained with secondary antibody only, ×40; **j** normal human duodenum, control stained with IgG instead of primary antibody, ×40. The *box* in the series of images to the *left*, a-c and d-f, represent the location of the magnified image to the *right*. Scale bar = 20 μm. *DCIS* ductal carcinoma in situ, *DCYTB* duodenal cytochrome b, *DMT1* divalent metal transporter 1, *ER* estrogen receptor, *PR* progesterone receptor

We observed that DCYTB was present on the luminal surface of epithelial cells in breast ducts and on the cell membrane of myoepithelial cells in normal breast tissue (Fig. 5a). Consistent with previous reports, erythrocyte membranes also stained positive for DCYTB [23]. Cribriform-type ductal carcinoma in situ (DCIS) showed intense staining along the luminal surfaces, similar to normal tissue, with additional faint cytoplasmic staining (Fig. 5b). Invasive tumors displayed reduced gland/tubule formation [45, 46], with a corresponding reduction in epithelial cells with membrane expression of DCYTB (Fig. 5c). Consistent with DCTYB mRNA levels, quantification of immunohistochemical staining in breast epithelial cells revealed that DCYTB protein was significantly reduced in invasive ductal carcinoma breast cancers (n = 60) as compared to normal adjacent breast epithelial tissue (n = 3) (*p* = .019, Additional file 1: Figure S8).

In addition to its role in intestinal iron uptake, DYCTB has been suggested to detoxify excess iron in bronchial epithelial cells through a mechanism involving DCYTB-mediated ferrireduction, uptake of divalent iron by divalent metal transporter 1 (DMT1), and storage in ferritin [22]. Since a role for DCYTB in either iron import or detoxification requires DMT1, we performed immunohistochemical analysis of DMT1. As expected, in control duodenal tissue, expression of DCYTB and DMT1 overlapped (Fig. 5g,h), consistent with the functional partnership of DCYTB and DMT1 in iron reduction and import in this tissue [16, 47]. In contrast, in the breast, expression of DMT1 was predominantly cytoplasmic, with minimal membrane staining (Fig. 5d-f). Collectively, these data suggest that DCYTB expressed in breast tissue may not function in its typical iron import role.

### Effects of DCYTB expression on iron metabolism in breast cancer cells

To directly test whether DCYTB affects iron metabolism in breast cells, we selected breast cell lines with high and low expression of DCYTB. As shown in Additional file 1: Figure S9, Western blot and qRT-PCR analysis indicated that T47D ductal carcinoma cells exhibited high basal expression of DCYTB, whereas MCF7 breast cancer cells exhibited substantially lower DCYTB expression.

**Fig. 6:**
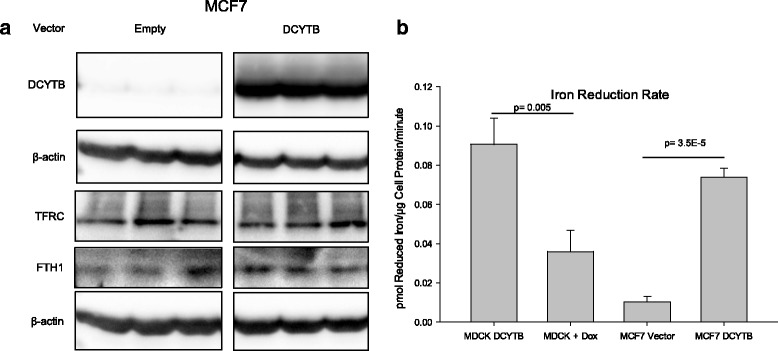
Expression and activity of DCYTB in cultured breast cells does not affect iron metabolism. **a** Western blot of iron-responsive protein expression in constitutive DCYTB expressing MCF7 cells. Triplicate samples are shown. **b** FerroZine assay at pH 6.4 of indicated cells. Results are the mean and standard deviation of triplicate samples. Similar results were obtained in at least three independent experiments. *DCYTB* duodenal cytochrome b

To determine whether DCYTB played a role in iron import in breast cancer cells, we overexpressed and knocked down DCYTB and assessed effects on parameters of iron metabolism. We first constitutively overexpressed DCYTB in MCF7 cells, which express low levels of endogenous DCYTB (Fig. 6a). To confirm that this exogenous DCYTB was functional, we measured its enzymatic activity using a ferrireductase assay. Tet-off DCYTB-EGFP MDCK cells, which have been previously shown to express doxycycline-regulated functional DCYTB with ferrireductase activity [18], were used as a control. As seen in Fig. 6b, MCF7 cells overexpressing DCYTB had significantly higher ferrireductase activity than cells transfected with empty vector. Control DCYTB-EGFP MDCK cells exhibited the expected doxycycline-regulated decline in ferrireductase activity (Fig. 6b). Thus exogenous DCYTB is expressed and functional in MCF7 cells.

We then tested whether DCYTB modulated iron import by examining transferrin receptor 1 (TFRC) and ferritin H (FTH1), two sensitive indicators of intracellular iron [48–50]. Expression of these proteins is posttranslationally regulated by iron: transferrin receptor expression is increased in iron deplete conditions and decreased in iron replete conditions, while the opposite is true of ferritin H. Thus, high TFRC expression coupled with low FTH1 is indicative of a state of decreased cellular iron, whereas low TFRC and high FTH1 indicates elevated levels of cellular iron. We observed no difference in transferrin receptor or ferritin H expression in MCF7 cells expressing DCYTB when compared to cells infected with the empty vector (Fig. 6a), indicating that exogenous DCYTB does not affect levels of intracellular iron.

**Fig. 7:**
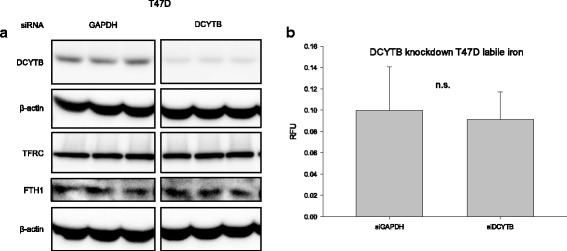
Knockdown of DCYTB in T47D cells does not affect proteins of iron metabolism. **a** Western blot of T47D cells with siRNA-mediated knockdown of DCYTB or GAPDH (control). Triplicate samples are shown. **b** Labile iron pool of DCYTB knockdown and control T47D cells. Results represent the mean and standard deviation of at least 14 replicate samples. Similar results were obtained in at least three independent experiments. *DCYTB* duodenal cytochrome b

**Fig. 8:**
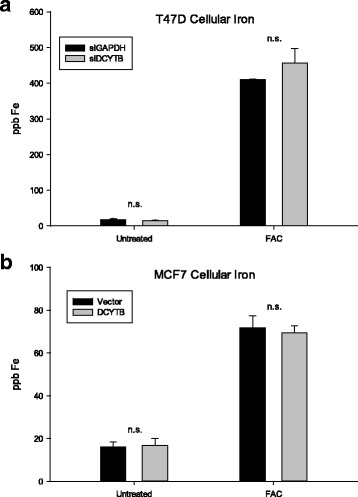
DCYTB expression does not affect total cellular iron. **a** ICP-MS analysis of total cellular iron in DCYTB knockdown T47D cells and **b** constitutive DCYTB-expressing MCF7 cells. Cells were either untreated or exposed to 200 μM ferric ammonium citrate (FAC) in growth medium for 24 hours. Results represent the mean and standard deviation of three replicates. *DCYTB* duodenal cytochrome b

To further investigate the effects of DCYTB, we performed the converse experiment by knocking down DCYTB in T47D cells, which express high levels of endogenous DCYTB (Fig. 7a). DCYTB was significantly reduced by transfection of targeted siRNA (Fig. 7a); however, ferritin H and transferrin receptor were not affected. Consistent with these results, measurement of the labile iron pool revealed no change in labile iron as a function of DCYTB expression (Fig. 7b). To confirm these results, we also assessed total cellular iron by inductively coupled plasma-mass spectrometry (ICP-MS) in DCYTB knockdown T47D cells and DCYTB-overexpressing MCF7 cells. Treatment with iron was used as a control. In both cell types, levels of intracellular iron were comparable, regardless of the level of DCYTB expression (Fig. 8a,b). This suggests that modulation of DCYTB expression does not significantly influence overall levels of cellular iron.

**Fig. 9:**
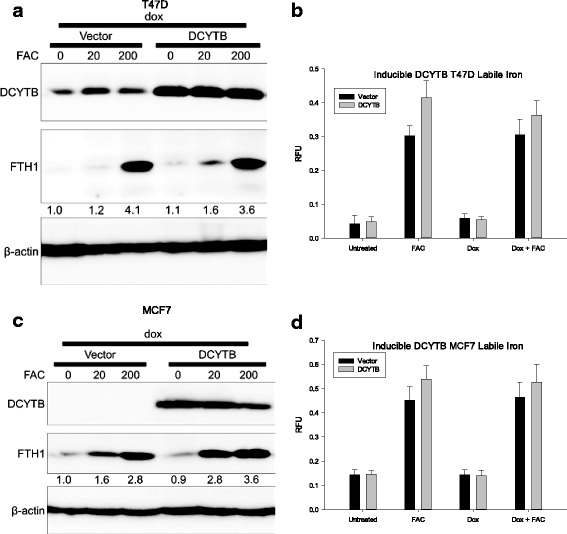
Iron-responsive protein expression and cellular labile iron in response to DCYTB induction. **a**. Iron-responsive protein expression in T47D cells induced with doxycycline for 72 hours. Transferrin receptor and ferritin expression quantified with Fiji ImageJ [83] and normalized to uninduced cells containing vector alone. **b** Labile iron pool measurement of T47D cells induced with doxycycline for 72 hours and iron treated for 24 hours. **c**. Iron-respnsive proein expression in MCF7 cells treated as in panel (**a)**. **d**. Labile iron pool measurements in MCF7 cells treated as in panel (**b**). Results represent the mean and standard deviation of at least 15 replicate samples. Similar results were obtained in at least three independent experiments. *DCYTB* duodenal cytochrome b

However, it remained possible that DCYTB might facilitate iron uptake under the specific condition of iron excess. To explore this, we used T47D and MCF7 cells expressing a Tet-inducible DCYTB expression vector, which enabled us to modulate DCYTB expression over a more graded range than that obtained using constitutive overexpression (Additional file 1: Figure S10). We found that in both T47D and MCF7 cells, basal levels of ferritin H were unaffected by DCYTB expression, regardless of the levels of DCYTB induction, supporting results obtained with constitutive expression of DCYTB (Additional file 1: Figure S10). We then compared the effect of DCYTB on the response of cells to exogenous iron (ferric ammonium citrate, FAC). In all cases, iron induced ferritin H and increased the labile iron pool to a similar extent (Fig. 9). Thus, in both T47D and MCF7 cells, there was an approximate three- to fourfold increase in ferritin with 200 μM FAC, regardless of the level of DCYTB (Fig. 9a and c). Similarly, labile iron in both T47D and MCF7 cells was unchanged by DCYTB expression (Fig. 9b,d). Consistent with these results, ICP-MS analysis of cells cultured for 24 hours in 200 μM FAC revealed no effect of DCYTB status on total cellular iron (Fig. 8a &b).

### DYCB inhibits adhesion and the activity of focal adhesion kinase

Although we observed that DCYTB was capable of reducing iron (Fig. 6b), expression of DCYTB had no measurable effect on iron levels in breast cancer cells (Figs. 6, 7, 8 and 9). We therefore sought to identify other molecular functions of DCYTB that might be responsible for its positive association with prognosis. To accomplish this, we compared expression profiles from patients that expressed the highest (≥90th percentile) and lowest (≤10th percentile) levels of DCYTB in cohorts #1 and #2 and then used the Signaling Pathway Impact Analysis (SPIA) package [51, 52] in the statistical software environment “R” [53] to discover pathways that might be affected by DCYTB expression. We found that expression of DCYTB was significantly associated with alterations in the cell cycle, focal adhesion, extracellular matrix (ECM)-receptor interaction and p53 signaling pathways (Additional file 2: Table S1). To test these associations experimentally, we first assessed the effect of knockdown or overexpression of DCYTB on cell proliferation and the cell cycle. We observed that the rate of increase in cell number was the same in T47D cells treated with siDCYTB or control siRNA, and was also unchanged in MCF7 cells that overexpressed DCYTB when compared to controls (Additional file 1: Figure S11a, b). Similarly, there was no effect of DCYTB on progression through the cell cycle in T47D cells treated with siGAPDH or siDCYTB (Additional file 1: Figure S12a, b). Thus, expression of DCYTB does not appear to directly affect cell cycle progression or proliferation of breast cancer cells.

**Fig. 10:**
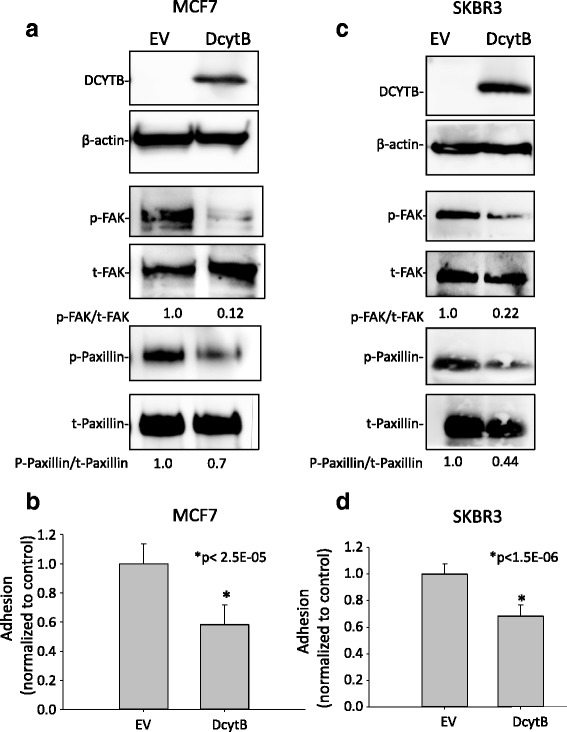
DCYTB expression inhibits adhesion to fibronectin. **a** Phosphorylation of FAK and paxillin was evaluated by Western blot in MCF7 cells expressing doxycycline-inducible DCYTB or control empty vector (EV). Quantification of staining intensity was performed using ImageJ. **b** Adhesion of MCF7 cells expressing DCYTB or control empty vector (EV) to fibronectin. **c** Phosphorylation of FAK and paxillin in SKBR3 cells expressing doxycycline-inducible DCYTB or control empty vector (EV). **d** Adhesion of SKBR3 cells expressing DYCTB or control empty vector (EV) to fibronectin. Graphs represent means and standard deviation of 16 replicates and are representative of three independent experiments. *DCYTB* duodenal cytochrome b

Given the association between DCYTB and the focal adhesion pathway found in the SPIA analysis, we next tested whether DCYTB affected focal adhesion kinase (FAK). FAK is a protein tyrosine kinase that plays a central role in regulating cell adhesion and motility, thereby promoting tumor progression and metastasis [54, 55]. We tested whether DCYTB affected FAK activation by measuring phosphorylation of FAK at tyr-925, a site that regulates focal adhesion turnover [56]. As seen in Fig. 10, MCF7 cells that expressed high levels of DCYTB exhibited substantially reduced FAK phosphorylation. Consistent with these results, DCYTB also reduced phosphorylation of paxillin, an adapter protein involved in maturation of focal adhesions [55] (Fig. 10). To directly assess the effect of DCYTB on adhesion, we measured the adherence of breast cancer cells to the extracellular matrix protein fibronectin. As seen in Fig. 10, DCYTB attenuated the ability of MCF7 cells to adhere to fibronectin. A reduction in FAK and paxillin phosphorylation and a corresponding inhibition of adhesion were also observed in SKBR3 breast cancer cells transfected with inducible DCYTB (Fig. 10). Collectively, these results indicate that DCYTB inhibits FAK activation and cell adhesion.

## Discussion

DCYTB was identified as one of 16 genes comprising an iron regulatory gene signature (IRGS) that is predictive of breast cancer patient survival [32]. In the IRGS, high expression of DCYTB was associated with improved distant metastasis-free survival. This was unexpected, because in the duodenum, DCYTB acts in conjunction with DMT1 to promote iron uptake, and an extensive literature links enhanced iron uptake with increased rather than decreased cancer risk [2–10]. Our results resolve this apparent paradox between the anticipated role of DCYTB and its association with favorable prognosis by revealing that in breast cancer cells, DCYTB does not play a role in iron acquisition.

We used immunohistochemical analysis to confirm the expression of DCYTB protein in breast tissue and to assess its cellular and subcellular localization (Fig. 5). We observed that DCYTB is present on the cell surface of epithelial and myoepithelial cells, and is particularly abundant at the luminal surface of ducts. DCYTB did not co-localize with DMT1, the transport protein with which DCYTB partners for uptake of iron, casting doubt on a role for DCYTB in iron transport or detoxification in breast cells (Fig. 5). We therefore used cell culture experiments to directly test the ability of DCYTB to impact iron metabolism in breast cancer cells.

Neither DCYTB overexpression nor DCYTB knockdown altered parameters of iron metabolism in breast cancer cells. Exogenously expressed DCYTB exhibited ferrireductase activity (Fig. 6b), indicating that the function of the transfected gene was preserved. However, basal levels of ferritin, an iron storage protein that is translationally regulated by iron, and transferrin receptor, an iron import protein that is posttranscriptionally regulated by iron, were unchanged following either overexpression of DCYTB in MCF7 cells (Fig. 6a) or knockdown in T47D breast cancer cells (Fig. 7a). Further, DCYTB overexpression did not affect the response of cells to excess exogenous iron (Figs. 8, 9a–c), the intracellular labile iron pool (Figs. 7b, 9b–d), or total cellular iron (Fig. 8).

To explore alternative roles for DCYTB in breast cancer, we used Signaling Pathway Impact Analysis (SPIA) as a discovery platform. We found that DCYTB exhibited a profound effect on the focal adhesion pathway, inhibiting phosphorylation of FAK, a kinase that regulates cell adhesion and motility [54, 57] and is often aberrantly expressed in cancer [58, 59] (Fig. 10). Phosphorylation of paxillin, an adaptor protein involved in maturation of focal adhesions, was similarly repressed by DCYTB, as was adhesion itself (Fig. 10). FAK lies at the center of a highly complex web of interacting proteins and signaling pathways [54, 55]. A connection between DCYTB and focal adhesions has not been previously observed, and further experiments will be required to elucidate the mechanism(s) by which DCYTB influences this complex pathway.

Consistent with an inhibitory role of DCYTB on FAK activation, analysis of two combined cohorts that together total 1610 breast cancer patients as well as the GOBO cohort (n = 1379) revealed that high DCYTB expression was associated with longer distant metastasis-free survival and longer relapse-free survival (both local and distant) (Fig. 1, Additional file 1: Figure S3).

Breast cancer patients have been successfully classified into outcome groups based on molecular profiling [37, 38], and several platforms for patient classification have been developed, including Oncotype Dx, Mammaprint, PAM50, and EndoPredict [60, 61]. DCYTB is not included in these currently available commercial and research-based classification systems. However, we observed that DCYTB expression increased in molecular subtypes with more favorable prognosis (Fig. 3 and Additional file 1: Figure S5), demonstrating that as a prognostic marker, DCYTB exhibits behavior that mimics known molecular markers of breast cancer.

Although evaluating patient prognosis is helpful to physicians and patients, predicting outcome of therapy is equally critical to clinical decision-making, and remains a challenge in breast cancer [60, 62, 63]. We therefore measured the association between DCYTB expression and survival in homogeneously treated groups of breast cancer patients [40, 64]. We used two cohorts: the first was a cohort of women with ER+ tumors who had been treated with tamoxifen monotherapy (Fig. 4a), and the second was a population of women with ERRB2- tumors treated with neoadjuvant chemotherapy (Fig. 4b). We observed a significant association of DCYTB expression with DMFS and relapse. In both cohorts, patients with low DCYTB expression were more likely to recur than those with high DCYTB expression (Fig. 4a,b). These results suggest that measurement of DCYTB expression may be useful in tailoring therapy: for example, it could help guide a subset of ER+ patients to more aggressive therapy, or alternatively, identify those for whom the risks of chemotherapy are less warranted. Use of gene expression to stratify breast cancer patients in this fashion has recently shown substantial promise [65].

## Conclusions

Our results demonstrate that DCYTB is a strongly associated with breast cancer patient prognosis and distinguishes disease outcome in homogeneously treated cohorts of breast cancer patients. Although DCYTB reduces iron and facilitates iron uptake in other tissues, in the breast, DCYTB functions via an iron-independent mechanism, attenuating activation of focal adhesion kinase and reducing cell adhesion.

## Methods

### Cell culture and reagents

Reagents were purchased from the following vendors: 17-β-estradiol (Sigma-Aldrich, St, Louis, MO, USA, E2758), Tamoxifen (4-hydroxy-(Z)) (EMD Millipore, Billerica, MA, USA, 579002), FerroZine (3-(2-Pyridyl)-5,6-diphenyl-1,2,4-triazine-p,p’-disulfonic acid monosodium salt hydrate) (Sigma-Aldrich, 160601), ferric ammonium citrate (Sigma-Aldrich, F5879), Doxycycline hyclate (Sigma-Aldrich, 9891), FuGENE® HD Transfection Reagent (Promega, Madison, WI, USA, E2311), hydroxyurea (Sigma-Aldrich, H8627). T47D breast cancer cells were obtained from the American Type Culture Collection (ATCC) and grown in RPMI-1640 basal medium containing 10% FBS at 37° in 5% CO_2_. MCF7 breast cancer cells were obtained from the ATCC and grown in EMEM containing 10% FBS and 10 U/ml insulin. MCF10A cells were obtained from the ATCC and cultured in MEGM containing MEGM Bulletkit™ with 100 ng/ml cholera toxin (Sigma-Aldrich, C8052). SK-BR-3 were purchased from ATCC and were grown in 10% FBS in HyClone™ McCoy’s 5A Media (GE Healthcare Life Sciences, Marlborough, MA, USA). MDCK cells were a generous gift of Dr. Andrew McKie and were cultured in DMEM supplemented with 10% Tet-free FBS (Takara Bio USA, Inc., Mountain View, CA, USA, 631106 or Fisher Scientific, SH3007003T) and puromycin (1.0 ng/ml) [18]. All basal media were obtained from Lonza (Basel, Switzerland). FBS was purchased from Gemini Bio-Products (Broderick, CA, USA).

### Construction and selection of cell lines with DCYTB overexpression

#### Constitutive DCYTB expression vector

The DCYTB coding sequencing was amplified from cDNA of U138MG cells and cloned into BamHI and XbaI sites of the pSL2 vector, a lentiviral overexpression vector containing enhanced green fluorescent protein (EGFP) [66]. Cloning primers were: DCYTB-F (5′ TCGGGATCCGCCATGGAGGGCTACTGGCGCT 3′) and DCYTB-R (5′ TAGTCTAGATCACATGGTAGATCTCTGCCCAG 3′). Sequence comparison with the reference gene in the NCBI database revealed that the cloned DCYTB cDNA was a polymorphic variant (S266N, rs10455 [67]). To express wild-type DCYTB, the mutation in the pSL2-DCYTB (S266N) variant was rectified using site-directed mutagenesis. All vectors were confirmed by DNA sequencing.

#### Inducible DCYTB expression vector

The following primers were used to amplify human DCYTB cDNA from pSL2-DCYTB plasmid: Forward (5′-CCCTCGTAAAGAATTCGCCACCATGGCCATGGAGGGCTACTGG-3′) and reverse (5′- GAGGTGGTCTGGATCCTTACATGGTAGATCTCTGCCCAGCC-3′). Primers contained restriction enzyme sites for EcoR1 and BamH1 respectively. The PCR product of DCYTB (861 bp) was digested with EcoR1 and BamH1 and inserted between the EcoR1/BamH1 sites of the pLVX-TetOne-Puro vector (Takara Bio USA, Inc., Mountain View, CA, USA). Plasmids were purified and sequenced. Cells were transfected using FuGENE® HD transfection reagent followed by 2 weeks of puromycin selection.

### siRNA

All reagents were obtained from GE Dharmacon (Lafayette, CO, USA) siDCYTB (D-17132-02 and D-17132-03) and siGAPDH (D-001140-01) were used for knockdown experiments. Transfections were performed according to the manufacturer’s recommendations using Dharmafect #1 (T-2001) transfection reagent.

### Western blotting

For DCYTB analysis, non-reduced samples were used; other samples were reduced. Cells were lysed in NP-40 lysis buffer (1% Nonidet P-40, 0.5% deoxycholate, and 0.1% SDS) in the presence of protease and phosphatase inhibitors (Roche Diagnostics, Basel, Switzerland) and proteins separated by SDS-PAGE. Western blots were probed with antibodies to DCYTB (Sigma-Aldrich, HPA014757), transferrin receptor (Thermo Fisher Scientific, Waltham, MA, USA, 13-6890), ferritin H [68], β-actin (Sigma-Aldrich, A3854), total FAK and P-FAK (Y925) (Cell Signaling Technology, Inc., Danvers, MA, USA, 13009 and 3284), phospho-paxillin (Cell Signaling Technology cat #2541), and paxillin (Cell Signaling Technology cat# 12065).

### mRNA expression

qRT-PCR was performed essentially as described [69], except that RNA was isolated and purified using the High Pure RNA Isolation Kit (Roche Diagnostics) and RT-qPCR was carried out using 2X SYBR® Green PCR Master Mix (Bio-Rad Laboratories, Inc., Hercules, CA, USA) in a ViiA7 cycler (Applied Biosystems, Inc., Foster City, CA, USA). Primers for PCR were designed with IDT PrimerQuest software (Integrated DNA Technologies, Inc., Coralville, IA, USA): DCYTB forward 5′-TGCATACAGTACATTCCCGCCAGA-3′, DCYTB reverse 5′-ATGGAACCTCTTGCTCCCTGTTCA-3′, ACTB forward 5′-TTGCCGACAGGATGCAGAAGGA-3′, ACTB reverse 5′-AGGTGGACAGCGAGGCCAGGAT-3′. GREB1 primers were as described in [70].

### Immunohistochemistry

Breast tissue microarrays were obtained from US Biomax, Inc., (Rockville, MD, USA). Antigen retrieval was performed using 0.05% citraconic anhydride (Acros Organics, Geel, Belgium) at pH 7.4 prior to immunostaining with a rabbit anti-DCYTB antibody (Sigma-Aldrich, HPA014757) or rabbit anti-DMT1 antibody (Sigma-Aldrich, HPA032140). Antibody to DCYTB was validated by immunofluorescence of cells that expressed high and low levels of DCYTB (Additional file 1: Figure S13). Slides were counterstained with hematoxylin (Poly Scientific R&D Corp., Bay Shore, NY, USA). Images were acquired using a Zeiss Axio Scan Z1 (Carl Zeiss Microscopy GmbH., Jena, Germany).To quantify DCYTB expression, stained microarray images were analyzed with Fiji software using reciprocal intensity as previously described [71]. Briefly, diaminobenzidine (DAB) signal was isolated from images by color deconvolution. Regions of interest were drawn around epithelial tissue throughout the entire tissue core. Mean DAB intensity/area was then measured in the regions of interest (breast epithelia). Reciprocal intensity (expressed in arbitrary units) was derived by subtracting the maximum intensity value from measured mean DAB intensity/area values.

### Immunofluorescence

4 × 10^5^ DCYTB or empty vector-expressing MCF7 cells were plated in an eight-chamber slide (BD Falcon, Franklin Lakes, NJ, USA). Cells were fixed with 4% paraformaldehyde for 15 minutes at room temperature, blocked with 5% BSA at room temperature for 2 hours, and incubated with anti-DCYTB (Sigma-Aldrich cat# HPA014757) antibody overnight at 4 °C. Alexa Fluor 555 conjugated anti-rabbit IgG secondary antibody was applied at 1:800 dilutions for 1 hour. Slides were mounted with ProLong Gold anti-fade reagent (Invitrogen, Carlsbad, CA, USA). Images were acquired using inverted microscopy (Zeiss Axio Vert.A1).

### Measurement of the labile iron pool (LIP)

The labile iron pool was measured essentially as described [72]. Briefly, cells were transfected with siRNA or treated with doxycycline for 48 hours. Cells were then transferred to 96-well plates and incubated for an additional 24 hours in growth medium with or without 200 μM ferric ammonium citrate (Sigma-Aldrich, F5879) for 4 or 24 hours prior to assay. Cells were washed, incubated with 2 μM calcein acetoxymethyl ester (Life Technologies, Carlsbad, CA, USA, C1430) for 15 to 30 minutes at 37 °C, washed with phenol-free EMEM, and 100 μM starch-conjugated deferoxamine (DFO) was added (a generous gift of Biomedical Frontiers, Inc., Minneapolis, MN, USA). Fluorescence was measured at 485 nm excitation and 535 nm emission (BioTek Synergy 2, BioTek, Winooski, VT, USA). Following stabilization of the fluorescence signal, 10 μM salicylaldehyde isonicotinoyl hydrazone (SIH) was added for several minutes until a stable signal was obtained. The change in fluorescence following the addition of SIH (ΔF) was used as a measure of the labile iron pool.

### Cell cycle analysis

Cells were synchronized with a 24-hour treatment of 2.0 mM hydroxyurea. Following release from synchronization, cells were removed from culture dishes and washed several times in PBS containing FBS and 2.0 mM EDTA and fixed in 70% ethanol at 4 °C overnight. Fixed cells were treated with RNase and stained with propidium iodide using FxCycle™ PI/RNase Staining Solution (Thermo Fisher Scientific, F10797). Fluorescence intensity was collected using a MACSQuant Analyzer (Miltenyi Biotec GmbH., Bergisch Gladbach, Germany). ModFit software (Verity Software House, Topsham, ME, USA) was used to calculate cell cycle histograms.

### Adhesion assay

MCF7 or SKBR3 cells containing empty vector or doxycycline-inducible DCYTB were treated with 1 μg/ml doxycycline for 72 hours, trypsinized, and 20,000 cells were allowed to adhere to a 96-well plate that had been coated with fibronectin (5 μg/ml). After 1.5 hours, cells were labeled with calcein-AM (Invitrogen), non-adherent cells were washed off, and adherent cells were quantified by measuring calcein fluorescence. Each experiment was repeated three times and 8–16 replicate wells were used in each determination. Significant differences were determined using two-tailed unpaired Student’s *t* tests.

### Microarray data sets

Cohort #1 was downloaded in October 2013 from Cancer Research [32] as a preprocessed file. Individuals with missing data (event data was unavailable for 18 patients) were excluded from the analysis. Cohort #2 was assembled from existing databases. Criteria for Cohort #2 were a median follow-up of greater than 2.5 years, greater than 100 patients in the study, an event rate of greater than 20% and gene expression analysis on the Affymetrix (Santa Clara, CA, USA) U133 platform and an outcome measure of recurrence-free survival. Four publicly available breast cancer patient datasets met our criteria: (i) 303 (Discovery, GSE25055) and 193 (Validation, GSE25065) patients from a prospective study at M.D. Anderson Cancer Center that identified a predictive signature of response to neoadjuvant chemotherapy [40]; (ii) a retrospective study of frozen tissue of 272 lymph node-negative patients from Rotterdam, Netherlands who did not receive systemic adjuvant or neoadjuvant therapy (GSE2034) [73]; and (iii) 101 cancer and 14 normal patient samples from Dublin, Ireland resected prior to hormone or chemotherapy (GSE42568) [74]. GSE25055 was downloaded April 2015 and GSE25065, GSE2034 and GSE42568 datasets were downloaded May 2015 from the National Center for Biotechnology Information Gene Expression Omnibus [75, 76] along with clinical and follow-up data. Where possible, CEL files were downloaded, preprocessed and RMA normalized. Surrogate variable analysis (SVA package) was used to batch correct cohort #2 [77, 78]. Analysis of the GOBO cohort was performed using online software (http://co.bmc.lu.se/gobo). Multivariable regression analysis was performed on patients for whom all variables were included in the dataset. This restricted analysis to 612 out of 1610 patients when comparing size, grade, age and ER status, and 464 patients when the analysis included LN status. A total of 571 patients were analyzed in the GOBO cohort.

### Statistical analysis

Analysis of microarray datasets was performed using R: A language and environment for computing using the affy [79], survival [80, 81], limma [82] and SPIA [51, 52] packages. Data downloaded for cohort #1 was on the Affymetrix U133A and B or U133plus2 platforms, on which two probes for DCYTB are present. In this case, the DCYTB probe with the highest absolute value of expression after normalization was used for downstream analysis. All data for cohort #2 was on the Affymetrix U133A platform, on which only one DCYTB probe is present. Kaplan-Meier (KM) survival analysis was used to determine distant metastasis-free survival (DMFS), relapse-free survival (RFS) (both local and distant) and bone-specific (RFS). Significance of KM plots was determined by the log-rank test. Cox proportional hazards regression was used to determine prognostic value of DCYTB when size, grade, age, ER status and LN status were included in the model. We used the Signaling Pathway Impact analysis (SPIA) algorithm [51, 52], implemented in R, to identify significantly activated or inhibited pathways [pFWER (family-wise error rate) < 0.05], using information from KEGG pathway annotations and differentially expressed genes (*p* < 0.05) between high and low DCYTB-expressing groups. Significance in cell culture experiments was assessed using two-tailed *t* tests, with *p* < 0.05 accepted as significant. Significance of DCYTB immunohistochemical staining was assessed using the Mann-Whitney rank sum test since the data were not normally distributed (Shapiro-Wilk test).

### ICP-MS

All containers used for sample digestion and preparation were pretreated with trace metal grade HNO_3_ to remove metal contaminations. Protein samples were digested in 100 μl HNO_3_ (trace metal grade, Fisher Scientific) in polypropylene reagent tubes (Sarstedt, Nümbrecht, Germany) in a heating block at 90 °C for 3 hours after which 100 μl of 10 M H_2_O_2_ (trace metal grade, Fisher Scientific) was added to the solution. The digested sample was further diluted to 2 ml total volume with 1% HNO_3_ and stored in precleaned polypropylene tubes until measurement. To ensure elemental recovery of >90%, NIST reference material (freeze-dried, powdered bovine liver, SRM 1577c) as well as the common elemental standard mix (VHG Labs, Inc., Manchester, NH, USA) were simultaneously digested by the same method. To determine background contamination from the tubes an empty tube was treated with 1 ml HNO_3_ and prepared concomitantly with the samples.

Inductively coupled plasma-mass spectroscopy (ICP-MS) analysis was performed using an Agilent 7700x equipped with an ASX 250 autosampler (Agilent Technologies, Santa Clara, CA, USA). The system was operated at a radio frequency power of 1550 W, an argon plasma gas flow rate of 15 L/min, Ar carrier gas flow rate of 1.04 L/min. Elements were measured in kinetic energy discrimination (KED) mode using He gas (4.3 ml/min). Data were quantified using a 9-point (0, 0.5, 1, 2, 5, 10, 50, 100, 1000 ppb (ng/g)) calibration curve with external standards for Mg, Mn, Fe, Cu, and Zn. For each sample, data were acquired in triplicate and averaged. A coefficient of variance was determined from frequent measurements of a sample containing 10 ppb of all elements analyzed. An internal standard (Sc, Ge, Bi) introduced with the sample was used to correct for detector fluctuation and to monitor plasma stability. Elemental recovery was evaluated by measuring NIST reference material (water SRM 1643e) and found to be >90% for all determined elements.

## Additional files

Additional file 1: Supplemental Figures.
**Figure S1.** DCYTB expression is higher in ER+ than ER- patients. **Figure S2.** DCYTB expression decreases with increased tumor grade. **Figure S3.** High DCYTB expression is associated with increased distant metastasis-free survival and reduced hazard in the GOBO combined breast tumor dataset. **Figure S4.** DCYTB predicts outcome independent of ER and LN status. **Figure S5.** Survival by molecular subtype in cohort #1. **Figure S6.** Increased DCYTB expression in molecular subtypes with better outcome in cohort #2. **Figure S7.** DCYTB expression is decreased in breast tumors. **Figure S8.** DCYTB protein is decreased in malignant breast tissue. **Figure S9.** DCYTB is expressed at higher levels in T47D cells than MCF7 cells. **Figure S10.** Induction and activity of Tet-on DCYTB expression vector. **Figure S11.** Modulation of DCYTB expression does not affect proliferation of cancerous breast cells. **Figure S12.** Knockdown of DCYTB expression does not affect progression through the cell cycle. **Figure S13.** Immunofluorescence staining of DCYTB or empty vector expressing MCF7 cells. (PPTX 4201 kb)
Additional file 2: Supplemental Table.
**Table S1.** Perturbed pathways identified by Signaling Pathway Impact Analysis (SPIA). (DOCX 15 kb)

## Abbreviations

DCIS: Ductal carcinoma in situ
DCYTB: Duodenal cytochrome b
DFO: Deferoxamine
DMFS: Distant metastasis-free survival
DMT1: Divalent metal transporter 1
EGFP: Enhanced green fluorescent protein
ER: Estrogen receptor
FAC: Ferric ammonium citrate
FTH1: Ferritin, heavy polypeptide 1
GAPDH: Glyceraldehyde 3-phosphate dehydrogenase
ICP-MS: Inductively coupled plasma-mass spectrometry
IRGS: Iron regulatory gene signature
LCN2: Lipocalin 2
LN: Lymph node
RFS: Relapse-free survival
SPIA: Signaling Pathway Impact Analysis
TFRC: Transferrin receptor 1
