# Supplementary material for: DCYTB is a predictor of outcome in breast cancer that functions via iron-independent mechanisms
Source: Breast Cancer Res. 2017 Mar 7;19:25. doi: 10.1186/s13058-017-0814-9 (PMC5341190; doi:10.1186/s13058-017-0814-9)
Supplement: Additional file 1: Supplemental Figures. — Figure S1. DCYTB expression is higher in ER+ than ER- patients. Figure S2. DCYTB expression decreases with increased tumor grade. Figure S3. High DCYTB expression is associated with increased distant metastasis-free survival and reduced hazard in the GOBO combined breast tumor dataset. Figure S4. DCYTB predicts outcome independent of ER and LN status. Figure S5. Survival by molecular subtype in cohort #1. Figure S6. Increased DCYTB expression in molecular subtypes with better outcome in cohort #2. Figure S7. DCYTB expression is decreased in breast tumors. Figure S8. DCYTB protein is decreased in malignant breast tissue. Figure S9. DCYTB is expressed at higher levels in T47D cells than MCF7 cells. Figure S10. Induction and activity of Tet-on DCYTB expression vector. Figure S11. Modulation of DCYTB expression does not affect proliferation of cancerous breast cells. Figure S12. Knockdown of DCYTB expression does not affect progression through the cell cycle. Figure S13. Immunofluorescence staining of DCYTB or empty vector expressing MCF7 cells. (PPTX 4201 kb) [file 13058_2017_814_MOESM1_ESM.pptx]

## Slide 1
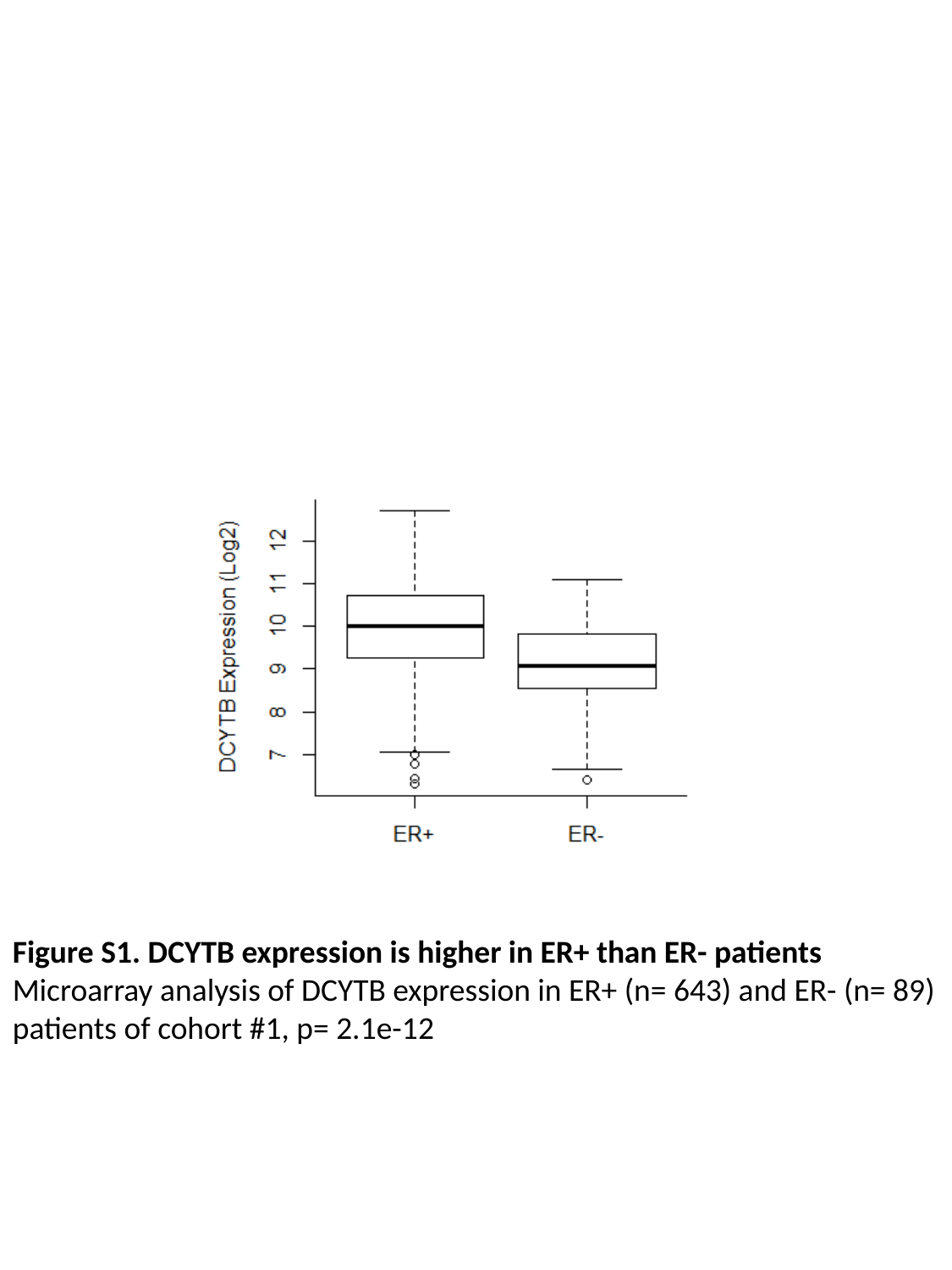

Figure S1. DCYTB expression is higher in ER+ than ER- patients
Microarray analysis of DCYTB expression in ER+ (n= 643) and ER- (n= 89) patients of cohort #1, p= 2.1e-12

## Slide 2
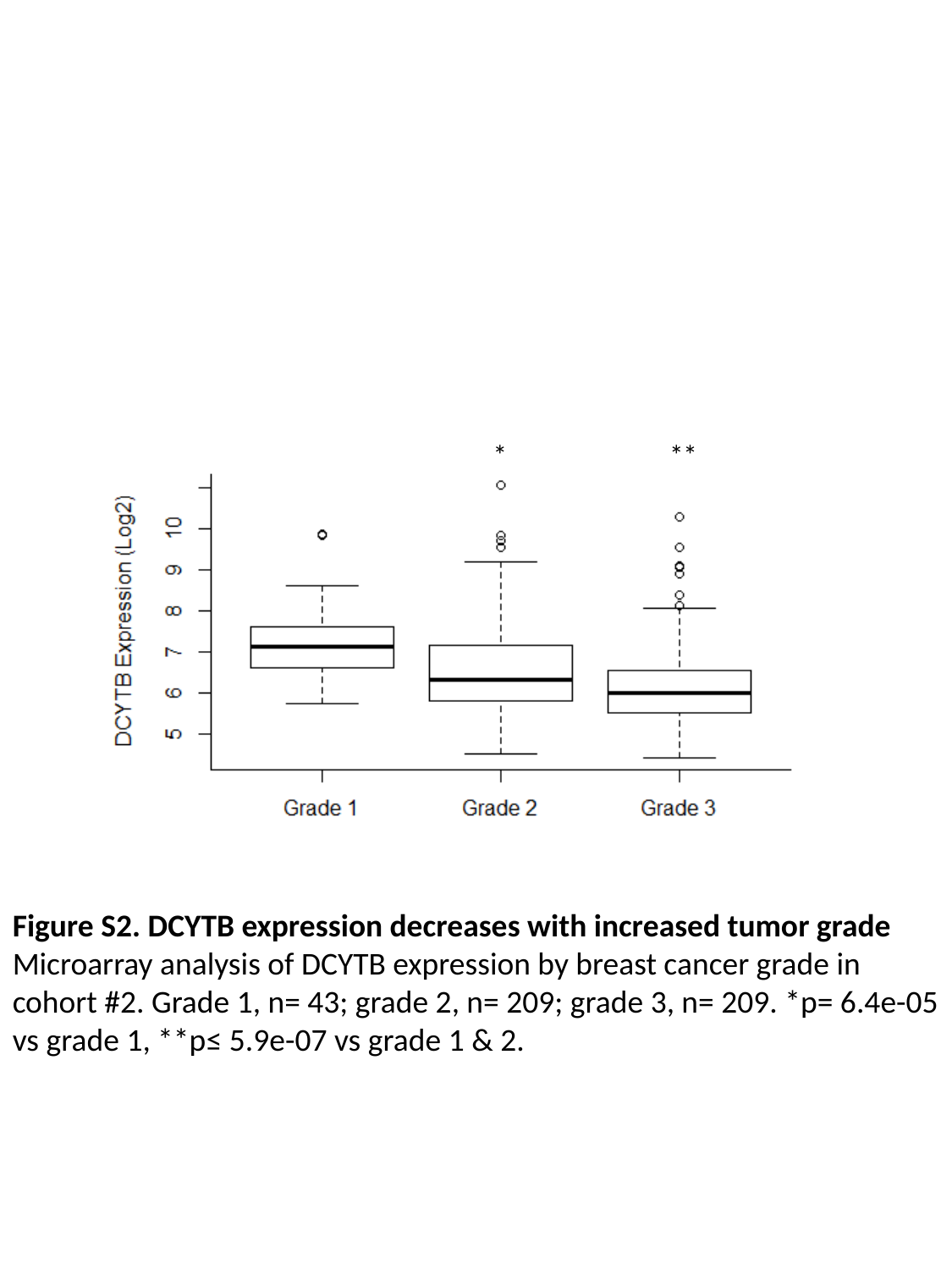

*
**
Figure S2. DCYTB expression decreases with increased tumor grade
Microarray analysis of DCYTB expression by breast cancer grade in cohort #2. Grade 1, n= 43; grade 2, n= 209; grade 3, n= 209. *p= 6.4e-05 vs grade 1, **p≤ 5.9e-07 vs grade 1 & 2.

## Slide 3
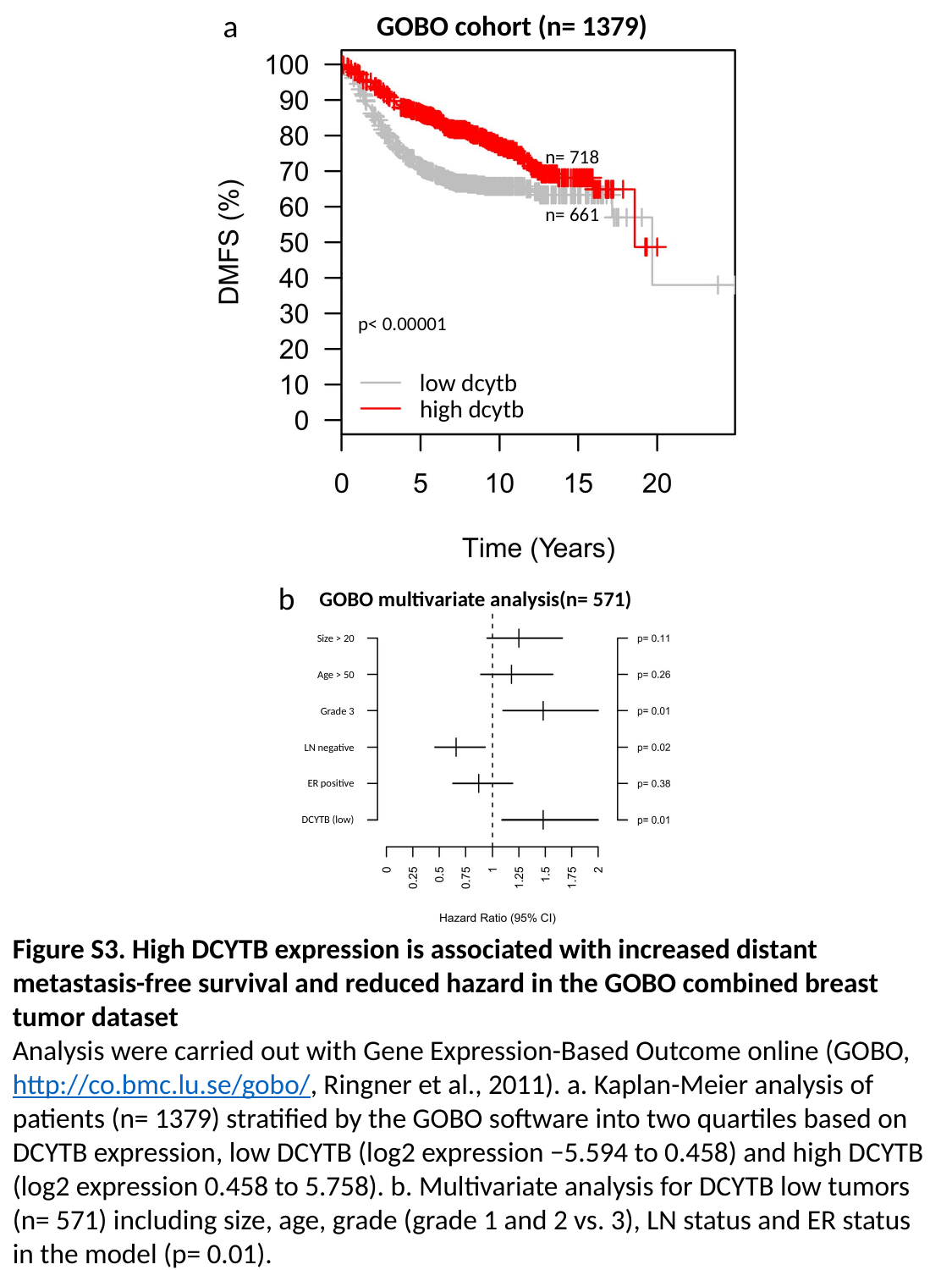

a
GOBO cohort (n= 1379)
n= 718
n= 661
p< 0.00001
low dcytb
high dcytb
b
GOBO multivariate analysis(n= 571)
Size > 20
Age > 50
Grade 3
LN negative
ER positive
DCYTB (low)
Figure S3. High DCYTB expression is associated with increased distant metastasis-free survival and reduced hazard in the GOBO combined breast tumor dataset
Analysis were carried out with Gene Expression-Based Outcome online (GOBO, http://co.bmc.lu.se/gobo/, Ringner et al., 2011). a. Kaplan-Meier analysis of patients (n= 1379) stratified by the GOBO software into two quartiles based on DCYTB expression, low DCYTB (log2 expression −5.594 to 0.458) and high DCYTB (log2 expression 0.458 to 5.758). b. Multivariate analysis for DCYTB low tumors (n= 571) including size, age, grade (grade 1 and 2 vs. 3), LN status and ER status in the model (p= 0.01).

## Slide 4
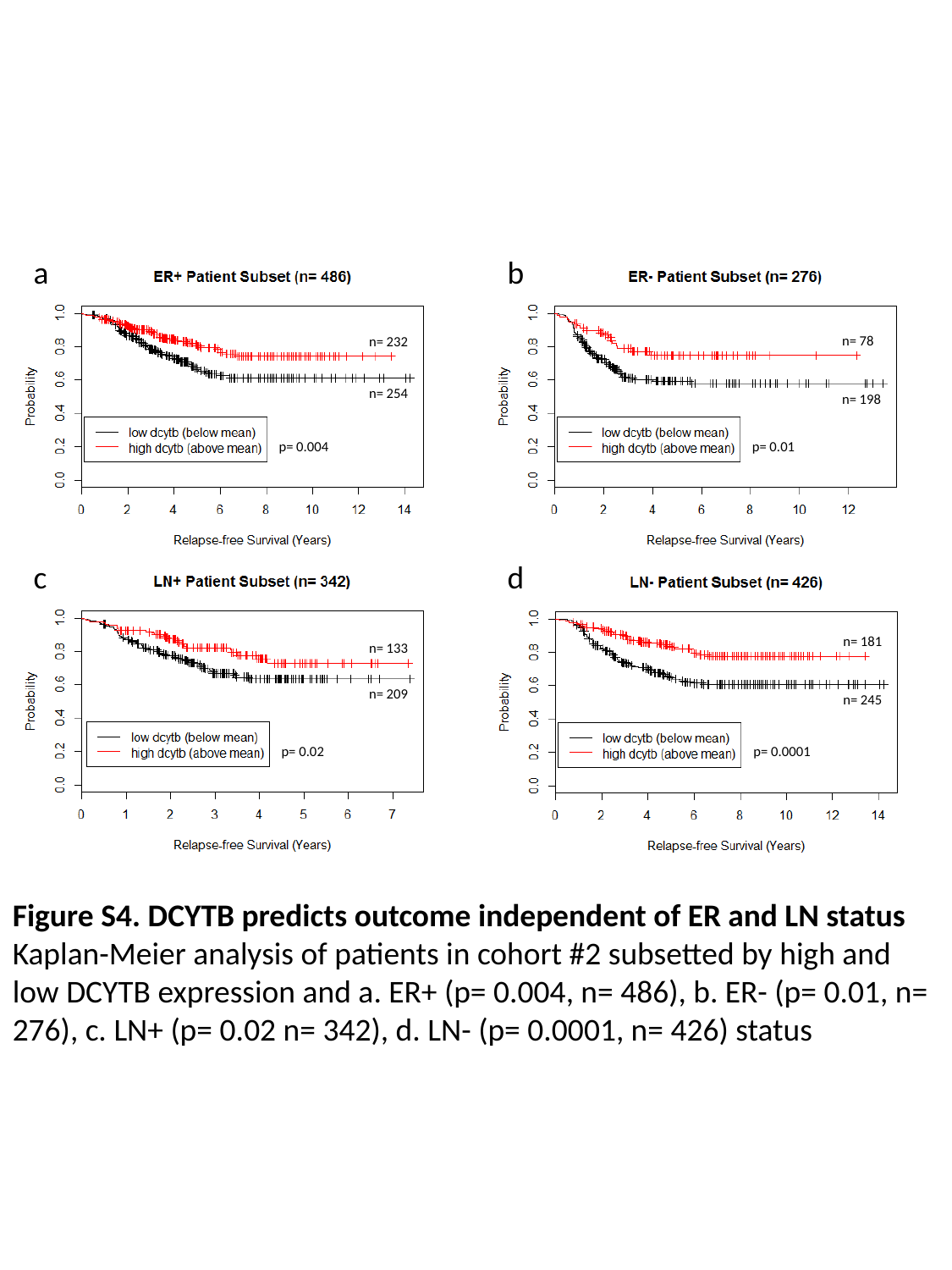

b
a
n= 78
n= 232
n= 254
n= 198
p= 0.01
p= 0.004
d
c
n= 181
n= 133
n= 209
n= 245
p= 0.02
p= 0.0001
Figure S4. DCYTB predicts outcome independent of ER and LN status
Kaplan-Meier analysis of patients in cohort #2 subsetted by high and low DCYTB expression and a. ER+ (p= 0.004, n= 486), b. ER- (p= 0.01, n= 276), c. LN+ (p= 0.02 n= 342), d. LN- (p= 0.0001, n= 426) status

## Slide 5
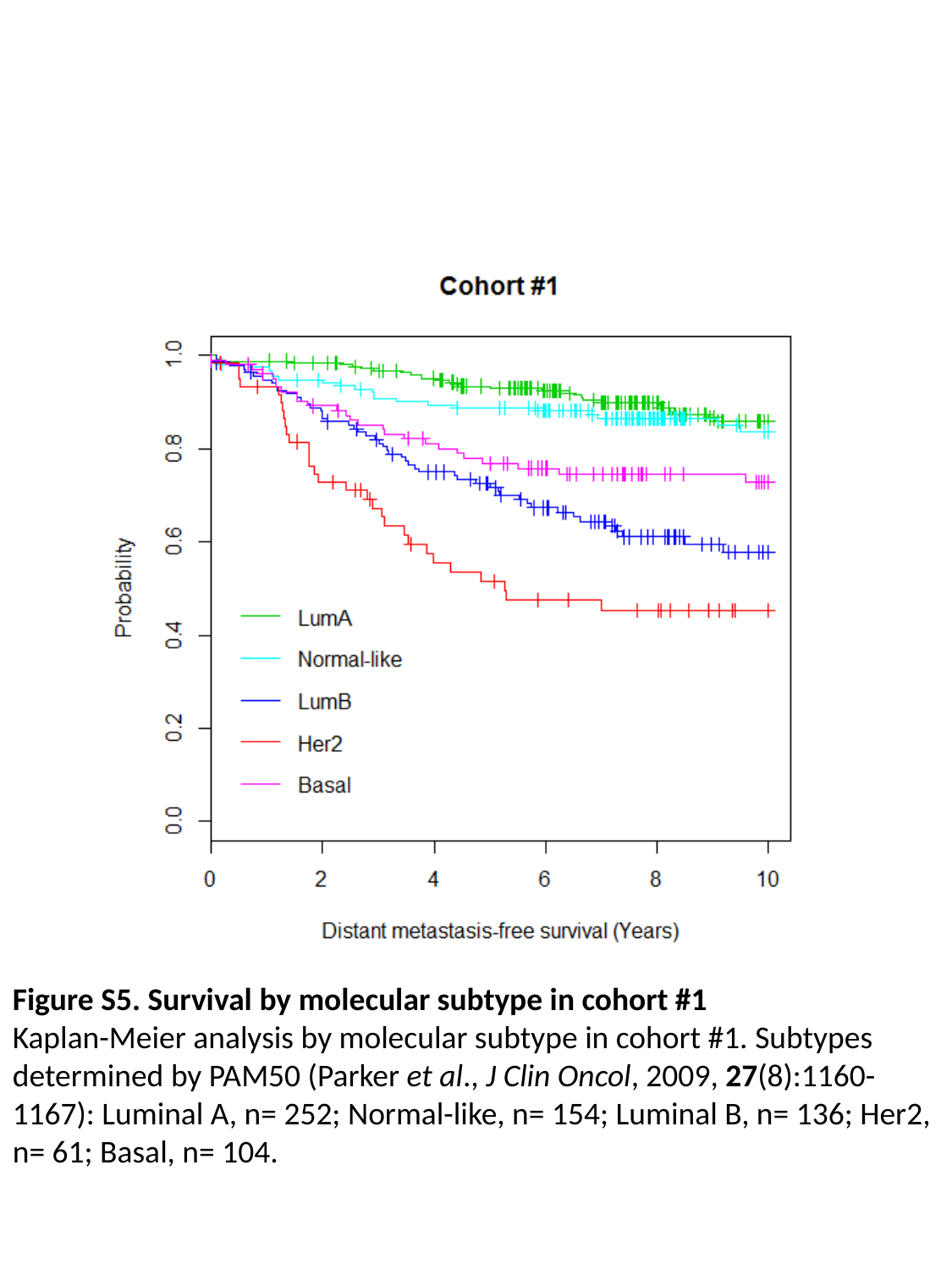

Figure S5. Survival by molecular subtype in cohort #1
Kaplan-Meier analysis by molecular subtype in cohort #1. Subtypes determined by PAM50 (Parker et al., J Clin Oncol, 2009, 27(8):1160-1167): Luminal A, n= 252; Normal-like, n= 154; Luminal B, n= 136; Her2, n= 61; Basal, n= 104.

## Slide 6
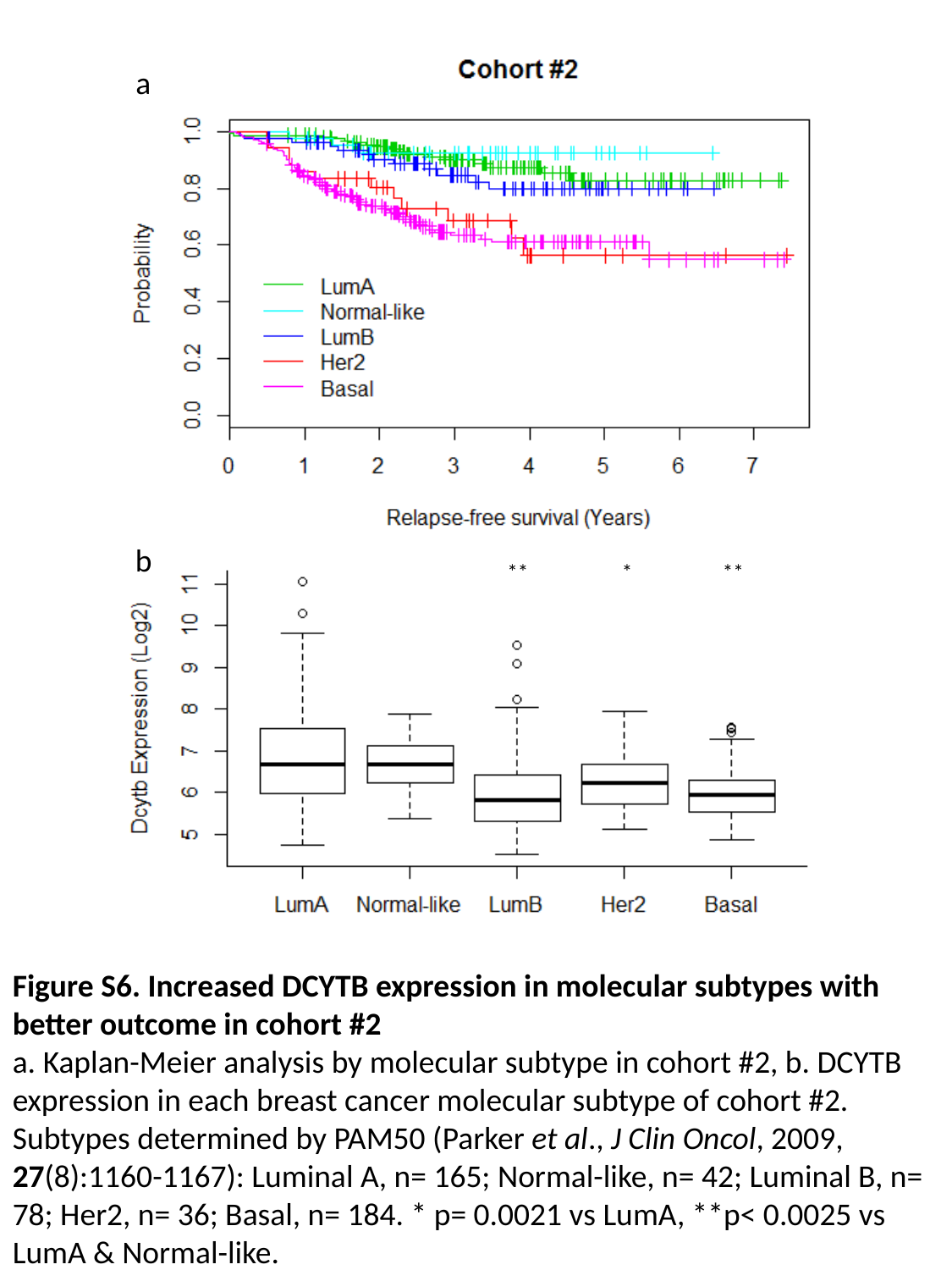

a
b
**
*
**
Figure S6. Increased DCYTB expression in molecular subtypes with better outcome in cohort #2
a. Kaplan-Meier analysis by molecular subtype in cohort #2, b. DCYTB expression in each breast cancer molecular subtype of cohort #2. Subtypes determined by PAM50 (Parker et al., J Clin Oncol, 2009, 27(8):1160-1167): Luminal A, n= 165; Normal-like, n= 42; Luminal B, n= 78; Her2, n= 36; Basal, n= 184. * p= 0.0021 vs LumA, **p< 0.0025 vs LumA & Normal-like.

## Slide 7
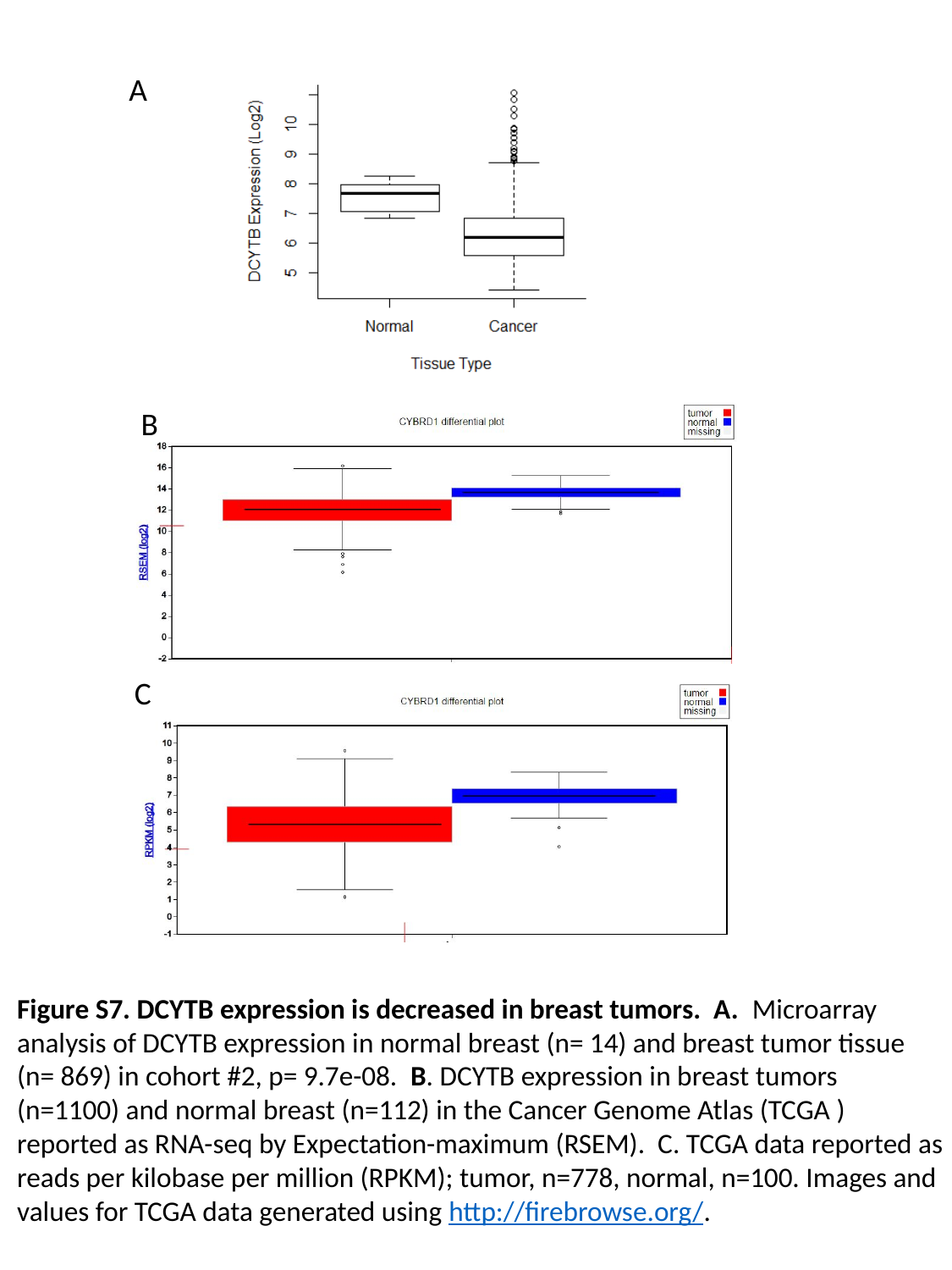

A
B
C
Figure S7. DCYTB expression is decreased in breast tumors. A. Microarray analysis of DCYTB expression in normal breast (n= 14) and breast tumor tissue (n= 869) in cohort #2, p= 9.7e-08. B. DCYTB expression in breast tumors (n=1100) and normal breast (n=112) in the Cancer Genome Atlas (TCGA ) reported as RNA-seq by Expectation-maximum (RSEM). C. TCGA data reported as reads per kilobase per million (RPKM); tumor, n=778, normal, n=100. Images and values for TCGA data generated using http://firebrowse.org/.

## Slide 8
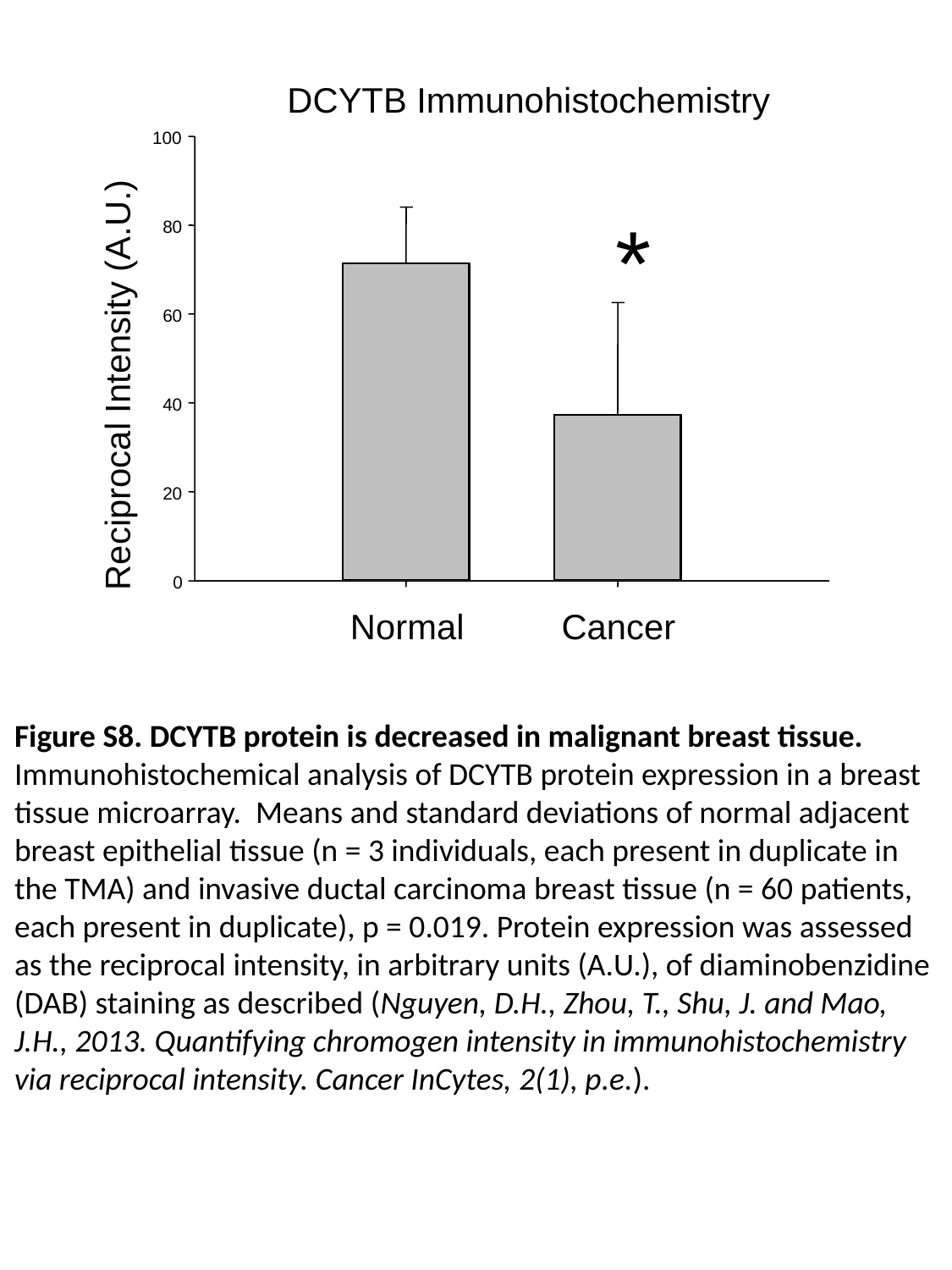

DCYTB Immunohistochemistry
100
*
80
60
Reciprocal Intensity (A.U.)
40
20
0
Normal
Cancer
Figure S8. DCYTB protein is decreased in malignant breast tissue. Immunohistochemical analysis of DCYTB protein expression in a breast tissue microarray. Means and standard deviations of normal adjacent breast epithelial tissue (n = 3 individuals, each present in duplicate in the TMA) and invasive ductal carcinoma breast tissue (n = 60 patients, each present in duplicate), p = 0.019. Protein expression was assessed as the reciprocal intensity, in arbitrary units (A.U.), of diaminobenzidine (DAB) staining as described (Nguyen, D.H., Zhou, T., Shu, J. and Mao, J.H., 2013. Quantifying chromogen intensity in immunohistochemistry via reciprocal intensity. Cancer InCytes, 2(1), p.e.).

## Slide 9
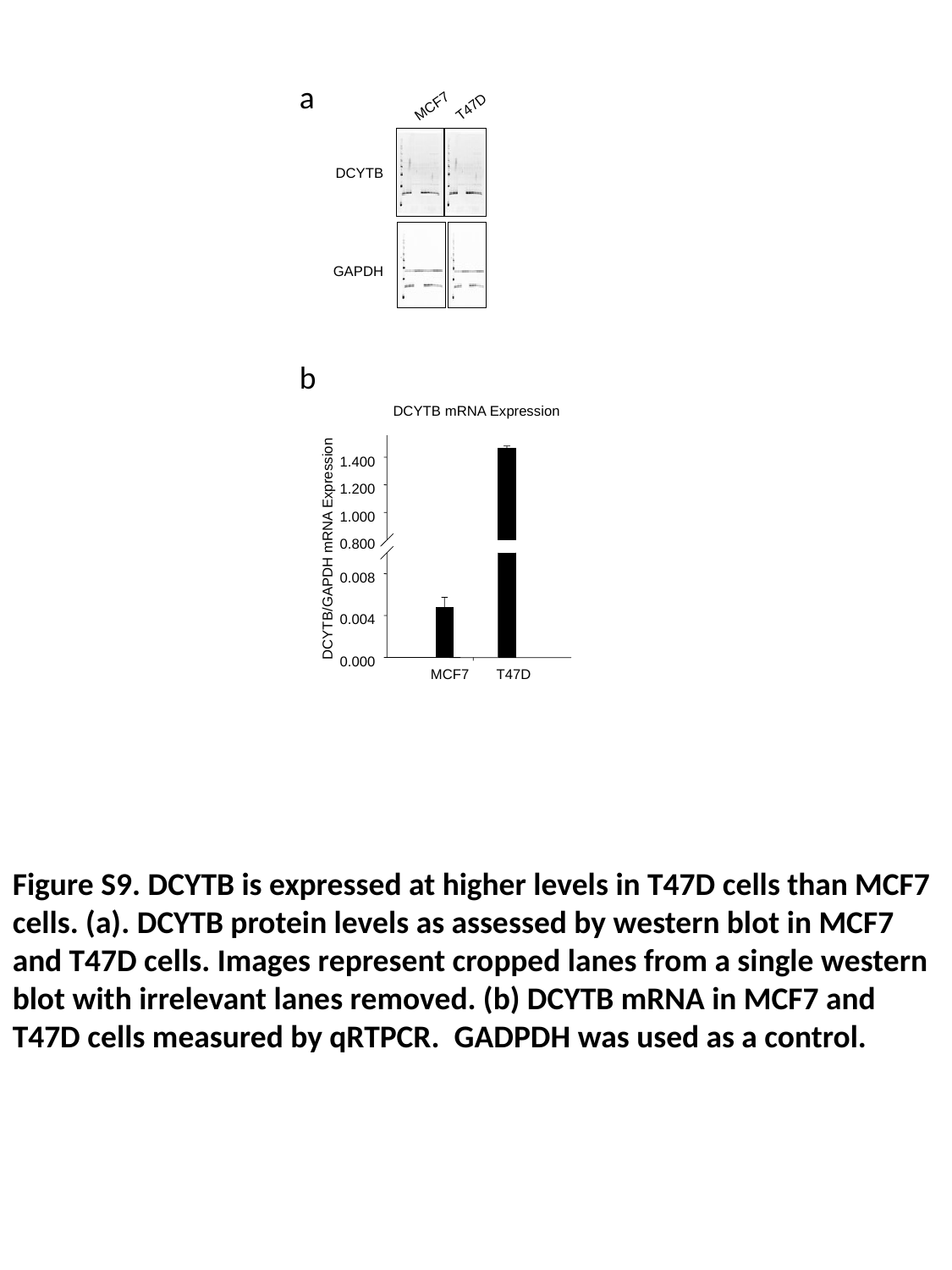

a
MCF7
T47D
DCYTB
GAPDH
b
DCYTB mRNA Expression
1.400
1.200
1.000
0.800
DCYTB/GAPDH mRNA Expression
0.008
0.004
0.000
MCF7
T47D
Figure S9. DCYTB is expressed at higher levels in T47D cells than MCF7 cells. (a). DCYTB protein levels as assessed by western blot in MCF7 and T47D cells. Images represent cropped lanes from a single western blot with irrelevant lanes removed. (b) DCYTB mRNA in MCF7 and T47D cells measured by qRTPCR. GADPDH was used as a control.

## Slide 10
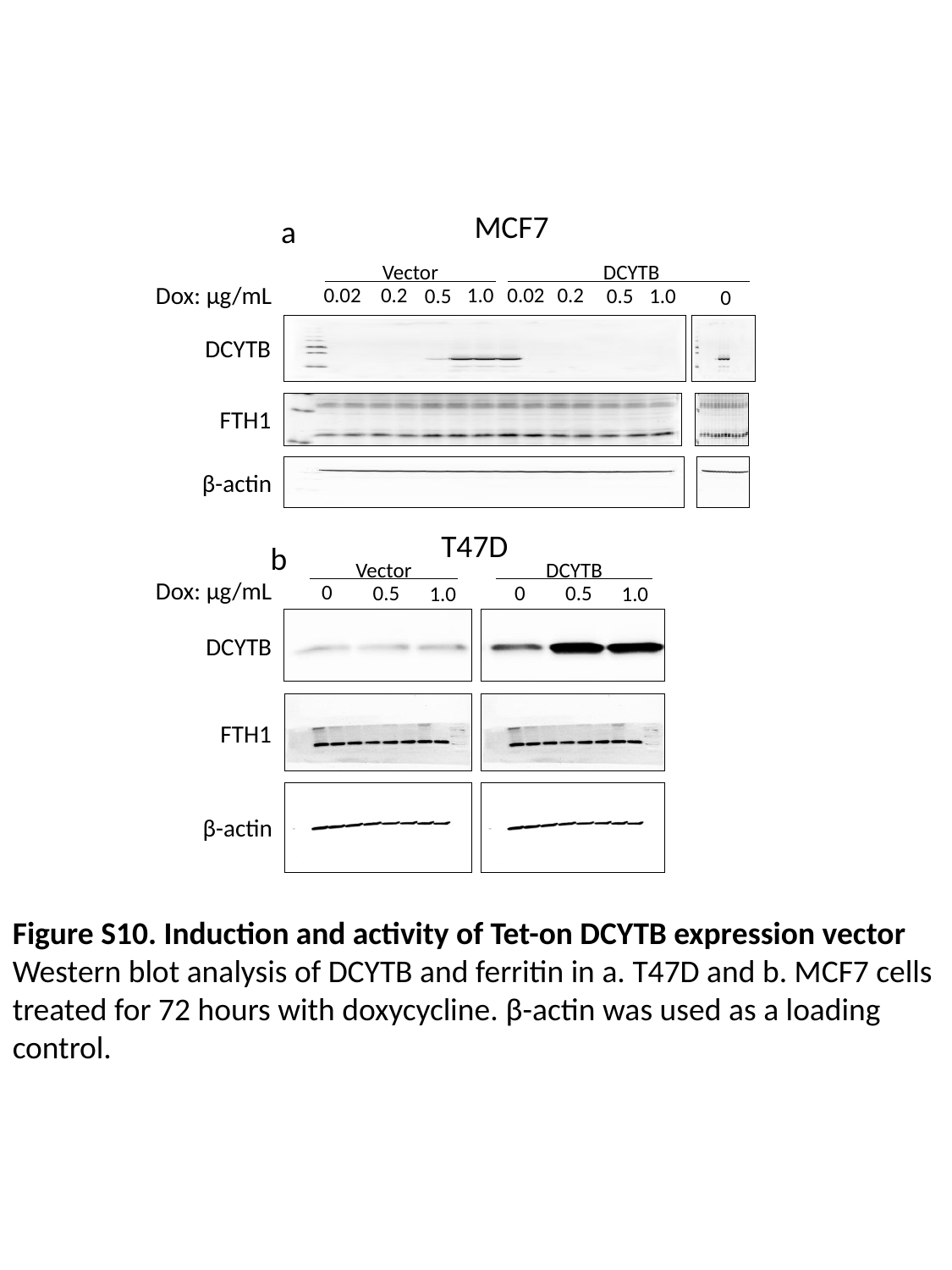

MCF7
a
Vector
DCYTB
Dox: µg/mL
0.02
0.02
0.2
0.2
1.0
0.5
0.5
1.0
0
DCYTB
FTH1
β-actin
T47D
b
Vector
DCYTB
Dox: µg/mL
0
0
0.5
0.5
1.0
1.0
DCYTB
FTH1
β-actin
Figure S10. Induction and activity of Tet-on DCYTB expression vector
Western blot analysis of DCYTB and ferritin in a. T47D and b. MCF7 cells treated for 72 hours with doxycycline. β-actin was used as a loading control.

## Slide 11
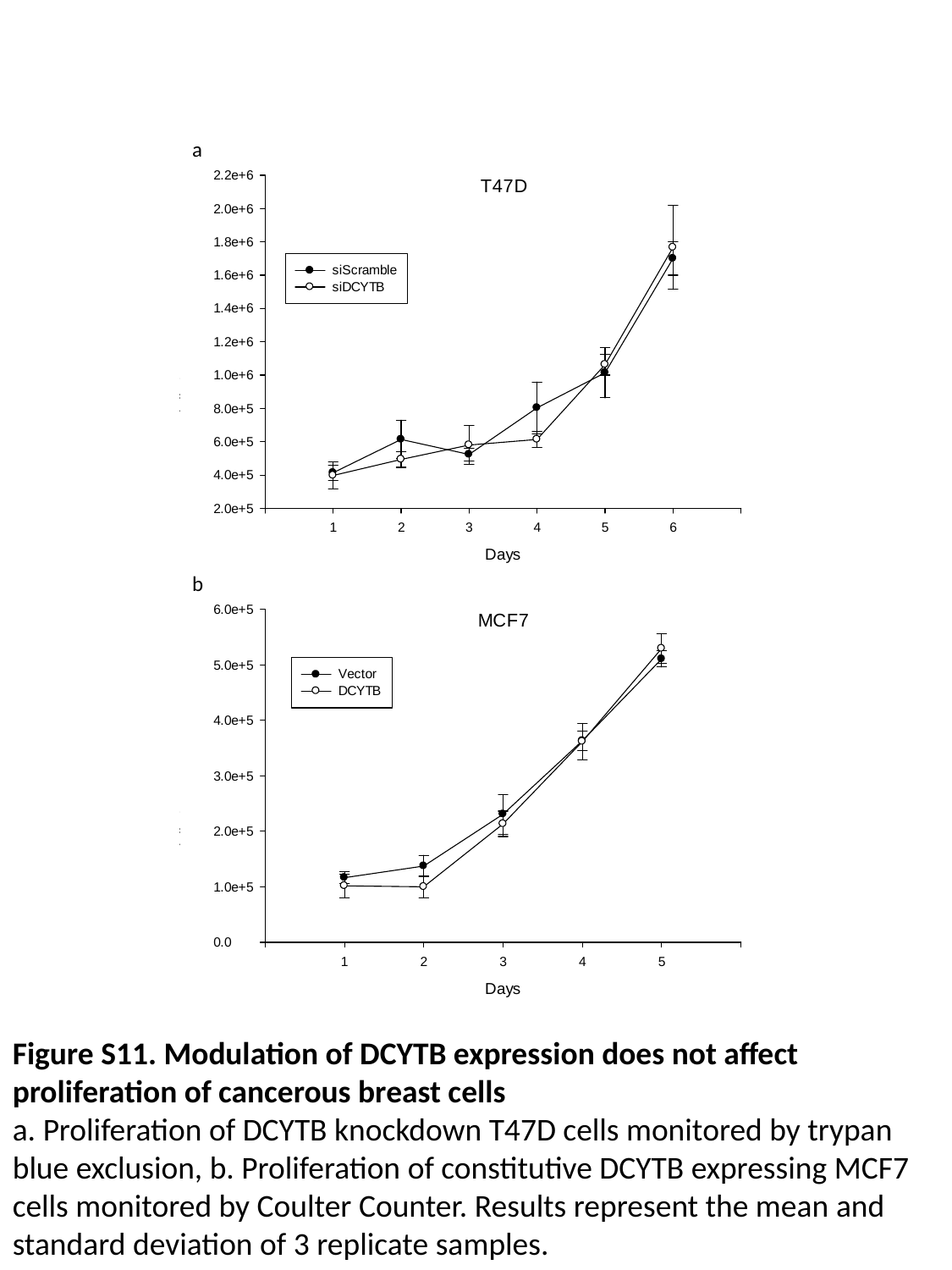

a
b
Figure S11. Modulation of DCYTB expression does not affect proliferation of cancerous breast cells
a. Proliferation of DCYTB knockdown T47D cells monitored by trypan blue exclusion, b. Proliferation of constitutive DCYTB expressing MCF7 cells monitored by Coulter Counter. Results represent the mean and standard deviation of 3 replicate samples.

## Slide 12
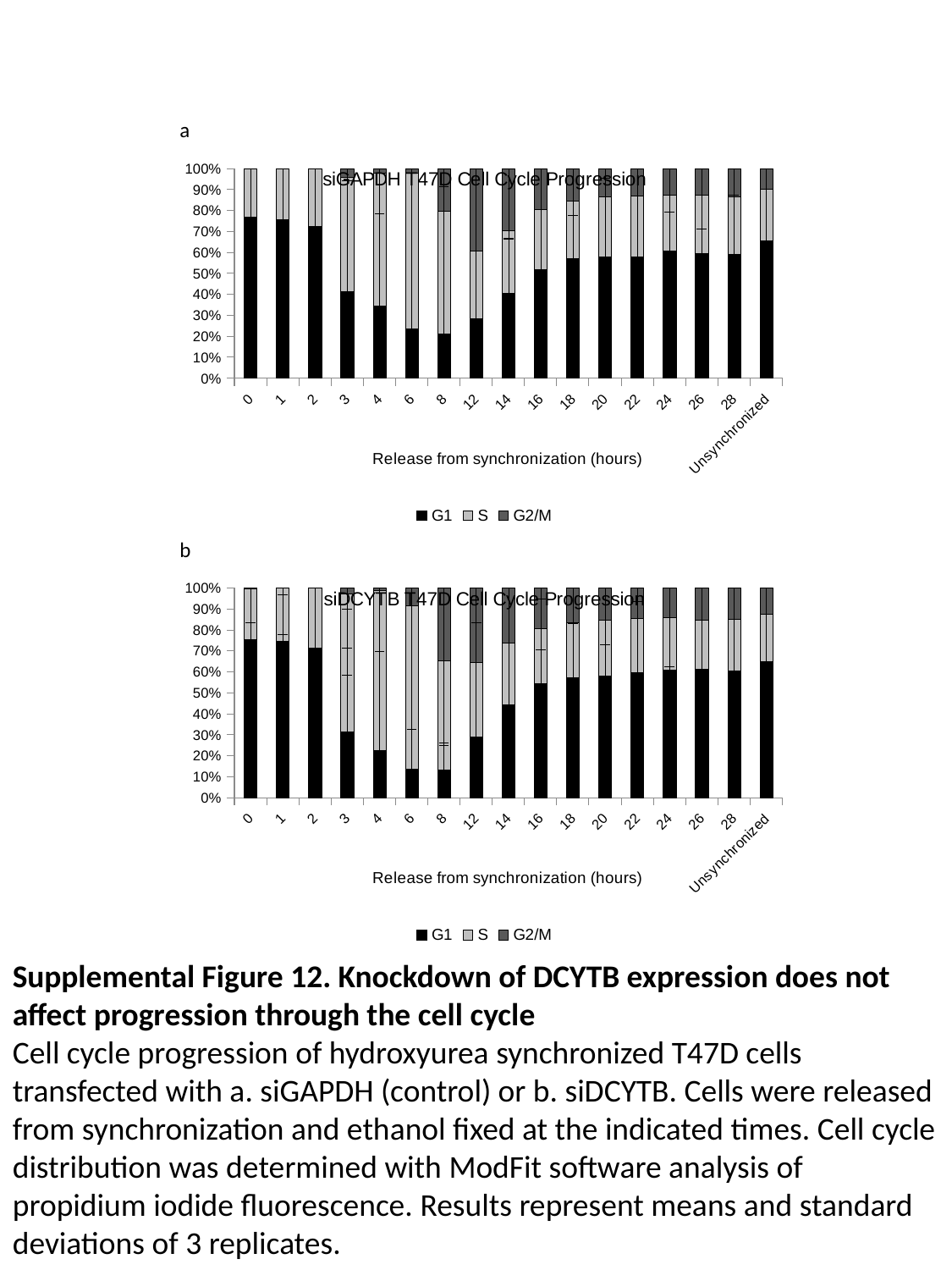

a
### Chart: siGAPDH T47D Cell Cycle Progression
| Category | G1 | S | G2/M |
|---|---|---|---|
| 0 | 76.78333333333333 | 23.21666666666667 | 0.0 |
| 1 | 75.57333333333334 | 24.426666666666666 | 0.0 |
| 2 | 72.32333333333332 | 27.676666666666666 | 0.0 |
| 3 | 41.48333333333333 | 54.52333333333333 | 3.9866666666666664 |
| 4 | 34.60666666666666 | 63.28 | 2.11 |
| 6 | 23.646666666666665 | 74.22666666666667 | 2.13 |
| 8 | 21.95 | 60.05666666666667 | 20.986666666666665 |
| 12 | 27.373333333333335 | 31.436666666666667 | 37.72666666666667 |
| 14 | 40.586666666666666 | 29.849999999999998 | 29.563333333333333 |
| 16 | 52.04333333333333 | 28.376666666666665 | 19.580000000000002 |
| 18 | 57.326666666666675 | 27.103333333333335 | 15.573333333333332 |
| 20 | 57.92333333333334 | 28.62 | 13.526666666666666 |
| 22 | 57.46666666666667 | 28.546666666666667 | 12.983333333333334 |
| 24 | 60.629999999999995 | 26.973333333333333 | 12.4 |
| 26 | 59.666666666666664 | 27.613333333333333 | 12.716666666666667 |
| 28 | 59.18666666666667 | 27.313333333333333 | 13.496666666666668 |
| Unsynchronized | 65.77333333333333 | 24.343333333333334 | 9.879999999999999 |b
### Chart: siDCYTB T47D Cell Cycle Progression
| Category | G1 | S | G2/M |
|---|---|---|---|
| 0 | 75.35333333333334 | 24.48 | 0.16666666666666666 |
| 1 | 74.75 | 25.25 | 0.0 |
| 2 | 71.29666666666667 | 28.703333333333333 | 0.0 |
| 3 | 31.613333333333333 | 65.66333333333333 | 2.723333333333333 |
| 4 | 22.599999999999998 | 76.22333333333333 | 1.18 |
| 6 | 13.486666666666666 | 78.09666666666668 | 8.416666666666666 |
| 8 | 13.413333333333332 | 51.97 | 34.620000000000005 |
| 12 | 29.11 | 35.339999999999996 | 35.553333333333335 |
| 14 | 44.23 | 29.75333333333333 | 26.016666666666666 |
| 16 | 54.376666666666665 | 26.276666666666667 | 19.343333333333334 |
| 18 | 57.330000000000005 | 26.073333333333334 | 16.596666666666668 |
| 20 | 58.22666666666667 | 26.526666666666667 | 15.243333333333334 |
| 22 | 59.596666666666664 | 25.76 | 14.64 |
| 24 | 61.04 | 24.826666666666668 | 14.166666666666666 |
| 26 | 61.31333333333333 | 23.546666666666667 | 15.14 |
| 28 | 60.379999999999995 | 24.88 | 14.733333333333334 |
| Unsynchronized | 65.16666666666667 | 22.21666666666667 | 12.616666666666665 |Supplemental Figure 12. Knockdown of DCYTB expression does not affect progression through the cell cycle
Cell cycle progression of hydroxyurea synchronized T47D cells transfected with a. siGAPDH (control) or b. siDCYTB. Cells were released from synchronization and ethanol fixed at the indicated times. Cell cycle distribution was determined with ModFit software analysis of propidium iodide fluorescence. Results represent means and standard deviations of 3 replicates.

## Slide 13
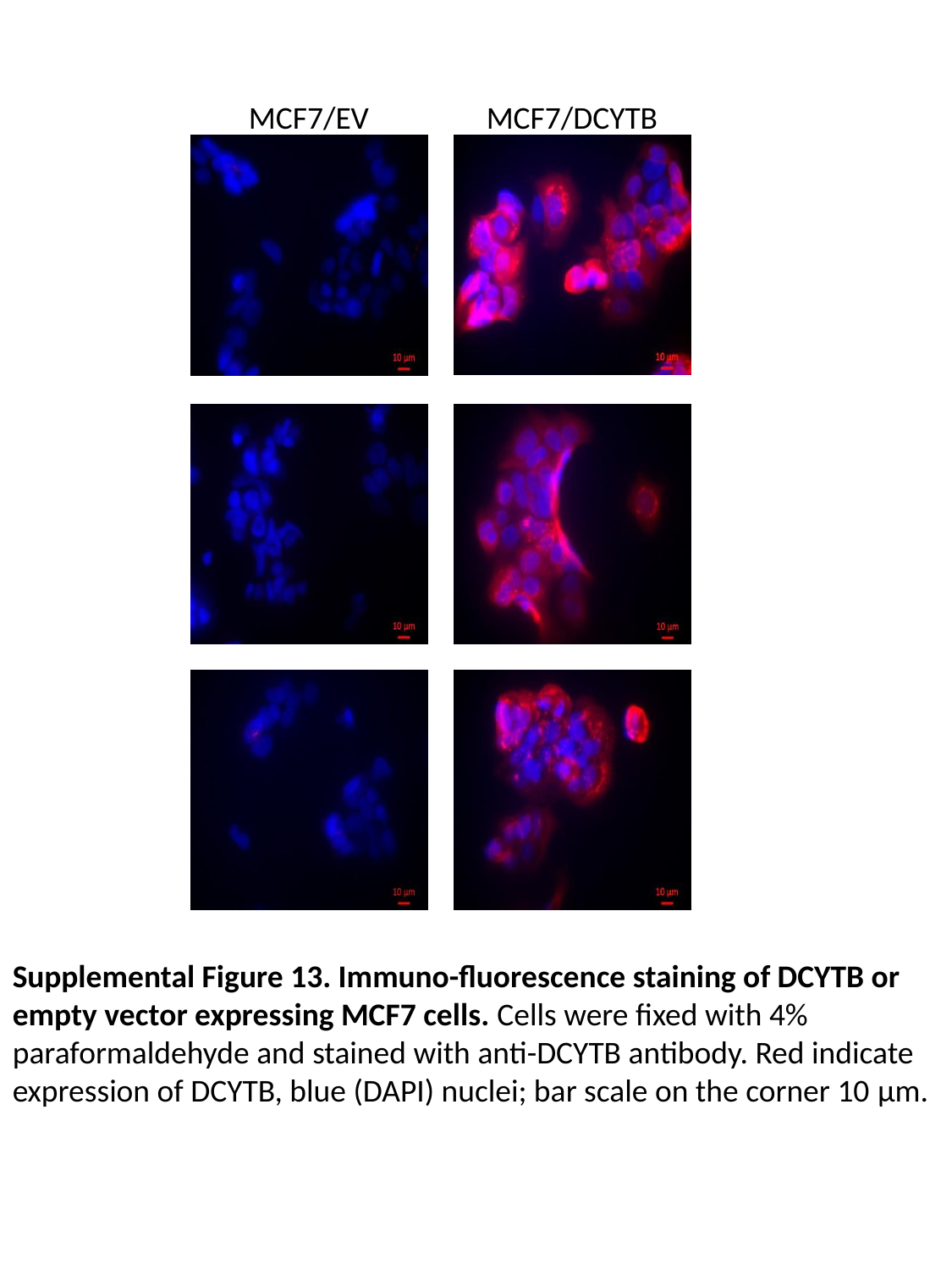

MCF7/EV
MCF7/DCYTB
Supplemental Figure 13. Immuno-fluorescence staining of DCYTB or empty vector expressing MCF7 cells. Cells were fixed with 4% paraformaldehyde and stained with anti-DCYTB antibody. Red indicate expression of DCYTB, blue (DAPI) nuclei; bar scale on the corner 10 μm.
