# Supplementary material for: DCYTB is a predictor of outcome in breast cancer that functions via iron-independent mechanisms
Source: Breast Cancer Res. 2017 Mar 7;19:25. doi: 10.1186/s13058-017-0814-9 (PMC5341190; doi:10.1186/s13058-017-0814-9)
Supplement: Additional file 2: Supplemental Table. — Table S1. Perturbed pathways identified by Signaling Pathway Impact Analysis (SPIA). (DOCX 15 kb) [file 13058_2017_814_MOESM2_ESM.docx]

**Table S 1. Perturbed pathways identified by Signaling Pathway Impact Analysis (SPIA)**

| **Pathway Name** | **KEGG Pathway ID** | **Differentially expressed genes** | **p-value** |
| --- | --- | --- | --- |
|  | | | |
| **Cohort #1 (n=152)** | | | |
| Focal adhesion | 4510 | 131 | 0.000178 |
| Cell cycle | 4110 | 93 | 0.000781 |
| ECM-receptor interaction | 4512 | 52 | 0.002462 |
|  |  |  |  |
| **Cohort #2 (n=174)** | | | |
| Cell cycle | 4110 | 63 | 0.000393 |
| p53 signaling pathway | 4115 | 23 | 0.039540 |

Data were subsetted into high and low DCYTB expression groups (> 0.9 quantile of expression, or < 0.1 quantile). The signaling pathway impact analysis (SPIA) algorithm (Tarca et al., 2009; Tarca et al., 2013, version 2.38), implemented in R, was used to identify significantly activated or inhibited pathways (pFWER< 0.05), using information from KEGG pathway annotations and differentially expressed genes (p< 0.05) between high and low DCYTB expressing groups.
